# Cerebrospinal fluid markers and magnetic resonance imaging lesion volume predicting relapse in canine meningoencephalitis of unknown origin

**DOI:** 10.3389/fvets.2026.1733620

**Published:** 2026-02-10

**Authors:** Franziska Spohn, Gloria Lesta, Adriano Wang-Leandro, Filip Kajin, Holger A. Volk, Jasmin N. Nessler

**Affiliations:** 1 Department of Small Animal Medicine and Surgery, University of Medicine Hannover, Foundation, Hannover, Germany; 2 DOS Software-Systeme GmbH, Wolfsburg, Germany; 3 Clinic for Internal Medicine, Faculty of Veterinary Medicine, University of Zagreb, Zagreb, Croatia

**Keywords:** canine, CSF-albumin, meningoencephalitis of unknown origin (MUO), MRI, relapse

## Abstract

**Introduction:**

Meningoencephalitis of unknown origin (MUO) is a potentially lethal neurological disease in dogs with a high relapse rate. Prognostic factors for relapse based on neurological examination, magnetic resonance imaging (MRI), or cerebrospinal fluid (CSF) examination are inconsistently reported.

**Methods:**

This retrospective single center study included 35 dogs with MUO. Brain MRI, CSF findings, clinical signs at diagnosis and during follow-up MRI (routine or relapse-related) were analyzed. Lesion volumes were calculated using the Cavalieri method. Relapse predictors were evaluated for routine follow- up MRI examinations using logistic regression and ROC/AUC.

**Result:**

Only higher CSF albumin (*p* = 0.0413) and lymphocyte proportion (*p* = 0.0288) at routine follow-up examination were predictive of future relapse. ROC analyses identified thresholds of 9.64 mg/dl for CSF albumin (AUC: 0.75; sensitivity 86.7%, specificity 61.1%) and 74.0% for CSF lymphocytes (AUC 0.76; sensitivity 66.7%, specificity 64.3%). Total lesion volume and volume of contrast-enhancing lesions decreased after treatment and increased again at relapse. Increased lesion volume or normal MRI on routine follow-up MRI did not reliably predict future relapse, although increased lesion volume in T1-weighted contrast enhancement and Fluid-attenuated inversion recovery (FLAIR) was observed at or after relapse. Lesion volume, lesion number, and lesion localization in different sequences was associated with neurodisability scale (NDS) and epileptic seizures.

**Discussion:**

In this study, CSF albumin and lymphocyte proportion were identified as predictors of future relapse. Routine follow-up MRI was not predictive of future relapse, but useful for detection of an active inflammation.

## 1 Introduction

Meningoencephalitis of unknown origin (MUO) is an umbrella term for a group of non-infectious inflammatory diseases of the central nervous system (CNS) in dogs (1). The clinical diagnosis is based on clinical signs of focal or multifocal lesions in the CNS, magnetic resonance imaging (MRI) findings, cerebrospinal fluid (CSF) pleocytosis, and the absence of detectable infectious diseases (2, 3). Nevertheless, the gold standard for diagnosis is histopathological diagnosis (4).

Immunosuppression with glucocorticoids remains the of cornerstone initial therapy, often in combination with other immunosuppressive drugs such as ciclosporin or cytarabine (5, 6). Please replace the sentence with: Nevertheless, 26%−33% of the dogs die despite therapy in the first 7 days after diagnosis (3, 7, 8). Of the dogs that survive, up to 65% experience a recurrence of clinical signs (3).

Several studies have been conducted to identify prognostic factors associated with improved outcomes in dogs diagnosed with MUO (5, 8). The occurrence of seizures or decreased mentation are clinical signs associated with a poorer prognosis in the first week after diagnosis (7, 9, 10). Furthermore, some studies have identified CSF parameters related to shorter survival time, such as an increased neutrophil cell count (7) and elevated CSF lactate levels (11). Additionally, MRI findings that may correlate with a poor short-term prognosis include loss of cerebral sulci, foramen magnum herniation, and mass effect (3, 12). Long-term prognosis might be worse with higher T2-weighted (T2w) lesion burden, and risk of a relapse seems increased with higher T1-weighted (T1w) post contrast lesion burden (8). A normal control MRI examination after 3 months is reported to be associated with a better outcome (3).

Repeated MRI examinations are helpful for monitoring dogs with MUO. Lowrie et al. (3), recommended a follow-up MRI examination 3 months after diagnosis, in combination with CSF analysis to evaluate the success of treatment. However, image interpretation is currently still purely subjective. In order to objectify this, recent studies have investigated quantitative approaches, such as measuring the total lesion burden (8) and assessment of brain atrophy based on the level of interthalamic adhesion in relation to brain volume (13), which may have a potential impact on prognosis.

The Cavalieri method is a well-established, reliable stereological measurement procedure. With this method it is possible to estimate the volume of three-dimensional structures on the basis of two-dimensional structures (14). One study showed that the Cavalieri method can estimate brain compartment volumes from MRI, with good agreement to postmortem reference measurements (15).

The aim of this study was to investigate MRI, CSF, and clinical findings in dogs with MUO during immunosuppressive therapy and to identify potential predictors of relapse. Focus was placed on longitudinal changes in lesion volume, the appearance of new lesions, CSF markers, and how these relate to neurological status and treatment decisions.

## 2 Materials and methods

### 2.1 Data collection

This is a retrospective, single-center cohort study of the Small Animal Hospital of the University of Veterinary Medicine Hannover including dogs presented for the diagnosis of MUO between 2013 and 2024. The medical records of the dogs were analyzed, and dogs were included in the study if the following criteria were met: more than one MRI examination, clinical signs suggestive of focal or multifocal CNS lesions, single or multiple intra-axial hyperintense lesions on T2w on the diagnostic MRI (present in all included dogs), initial CSF examination showing an increased cell count, exclusion of possible infectious agents (2), and if treatment with immunosuppressive therapy resulted in initial clinical improvement. Based on available records, no dogs with a normal diagnostic MRI underwent a second MRI examination and therefore did not meet the inclusion criteria.

Collected data included age, weight, sex, neuter status, breed, date of death, cause of death, number and date of examinations, occurrence of generalized tonic-clonic and focal seizures, status epilepticus and cluster seizures, results of the CSF examination (protein mg/dl, albumin in mg/dl, Qalb (ratio of CSF albumin and albumin of the blood) [commercially available photometric assay test adapted for dogs, cobas c311 analyzer, Hitachi, Roche, Mannheim, Germany], amount of white blood cells/3 μl (WBC), proportion of lymphocytes in %, segmented nucleated neutrophil granulocytes in %, and macrophages in %, treatment (immunosuppressive drugs, dosage, and change in medication including add-on therapy). Recorded neurological signs were used to determine the neurodisability scale (NDS) (1). Although retrospective assessment of the NDS may be less reliable than prospective application (1, 16). The NDS was used as a standardized clinical assessment tool to enable consistent comparisons across neurological examinations. This approach also allowed a uniform definition of clinical relapse. Relapse was defined as an increased of NDS at a follow-up examination compared with the preceding examination.

### 2.2 MRI evaluation

MRI examinations were performed using a 3.0 Tesla MRI Scanner (Achieva, Philips Medical Systems, Best, The Netherlands). Transverse T2w, FLAIR and T1w after intravenous injection of gadolinium-based contrast agent (Gadoteric acid; - Dotarem^®^, Guerbet GmbH Sulzbach, Germany) were obtained.

To objectify MRI findings, volume, quantity, and localization of the lesions was recorded. Lesion localization was recorded as within the brainstem, the forebrain (including: the hippocampus, the cingulate gyrus, the piriform lobe, the frontal lobe), the cerebellum, and the meninges. Furthermore, caudal transtentorial herniation, loss of sulci, or the presence of new lesions compared to the last MRI were noted.

All MRI evaluations (including lesion identification, measurements, and lesions volume quantification) were performed by the first author not blinded to the clinical information.

As a parameter to assess brain atrophy, the interthalamic adhesion height was measured on sagittal T2w sequence. Measurements were performed on the slice showing the maximum interthalamic adhesion height (17). A commercially available DICOM viewer was used for this purpose (Easy Image; VetZ GmbH Isernhagen, Germany).

For brain volume analysis an artificial intelligence (AI) based brain extraction was used to first create a 3D volume mask of the brain parenchyma by stripping the skull (18) from T2-weighted images: the DICOM sequences are read by the AI and converted into a 3D image with isometric voxels. Based on 3D model, the AI uses segmentation to create a mask in Nifti format, which distinguishes brain areas from other image content (18). The generated brain masks were saved and imported into 3D Slicer, where they were reviewed, manually corrected if necessary by the first author, and used for final brain volume calculation with Open-Source-Software 3D Slicer [version 5.8.0 for macOS from the National Institutes of Health (NIH) was used; https://www.slicer.org].

Lesion volume was determined using the image editing program ImageJ [National Institute of Health (NIH), based in Bethesda, Maryland USA] (19). Each lesion was manually outlined for every MRI in the transverse T2w, FLAIR and T1w post contrast images. A cross-counting grid (between 12 and 24 mm^2^) was applied, and grid points located within a brain lesion were counted for each slices.

With the total of the crosses for each brain lesion the volume of each lesion was determined using the Cavalieri Method. The lesion volume was estimated with the following formula as used in Watson et al. (14):


$$\begin{array}{l} \hat{V}=\;A_{p}m^{\prime }\bar{t}\left[\overset{n}{\underset{i=1}{\sum }}P_{i}\right] \end{array}$$

Ap is the area that each point represents. *m*′ represents the spacing between MRI slices. $\bar{t}$ represents the thickness of cross section (if every MRI sequences were counted would be one). *P*_*i*_ represents the number of grid points counted (14).

Total lesion volume was defined as the sum of the volume of all lesions per MRI sequence. The relative lesion volume was calculated as the ratio of total lesion volume to brain volume.

### 2.3 Data analysis

Data were analyzed using the statistical software SAS Studio Enterprise, Version 3.8.2 (SAS Institute Inc., Cary, North Carolina, USA).

For statistical analysis, data were first grouped according to MRI examination timepoint (first to fourth examination) to find differences in clinical, CSF, and MRI variables over repeated examinations. Dogs were additionally classified into relapse-related groups (before relapse, no relapse, relapse and after relapse) to identify differences in routine follow-up examinations and examinations at or after relapse.

This grouping approach was used to evaluate whether findings obtaining routine follow-up MRI examinations differed between dogs that subsequently relapsed and dogs that did not relapse.

To evaluate whether findings from routine follow-up MRI examinations were predictive of future relapse, MRI examinations performed at diagnosis (first MRI) and those conducted at or after relapse were excluded from this analysis. The remaining routine follow-up examination were compared between dogs that subsequently relapsed and dogs that did not relapse.

For all statistical analyses, a *p*-value ≤ 0.05 was considered statistically significant. Continuous variables were summarized as mean ± standard deviation (SD) for normally distributed data, or as median and interquartile range (IQR) for non-normally distributed data. Categorical variables were described as frequencies with 95% confidence intervals (CI).

Normality was assessed using the Shapiro–Wilk test and by visual inspection of histograms and boxplots. Correlations between continuous variables were examined using Pearson's correlation coefficient when both variables were normally distributed; otherwise, Spearman's rank correlation coefficient was used.

Linear regression was performed to explore associations between continuous variables. To identify predictors of relapse, binary logistic regression was conducted. Variables were selected based on univariable screening and clinical relevance. Specifically, variables that were statistically significant in univariate analyses were considered for inclusion; in addition, variables that were considered clinically relevant were also included regardless of the univariate *p*-value, as they may be important indicators of disease activity and are commonly used in clinical decision-making. Results are reported as odds ratios (OR) with corresponding 95% CIs. Receiver operating characteristic (ROC) analyses were additionally performed for CSF albumin and CSF lymphocytes. The optimal cut-off was determined using the Youden index, which selects the threshold with the highest combined value of sensitivity and specificity.

### 2.4 Ethic committee

As this was a retrospective study, no ethical approval was required in accordance with local legislation and institutional requirements. Only previously collected data were used. Written informed consent for the use of these data was obtained from the owners prior to the examinations, in accordance with the University's guidelines.

## 3 Results

A total of 35 dogs were included in the study. Of these, 18 dogs (51%) were male (14/18 intact and 4/18 neutered). The remaining 17 dogs (49%) were female (10/17 intact and 7/17 neutered). The following breeds were included: chihuahua (*n* = 5), French Bulldog (*n* = 5), mixed breed (*n* = 5), Labrador Retriever (*n* = 2), Jack Russel Terrier (*n* = 2), Yorkshire Terrier (*n* = 2) and one each Elo, Airedale Terrier, Australian Shepherd, Beagle, Biewer Terrier, Bolognese, Golden Retriever, Havanese, Pug, Pygmy Spitz, Small Münsterländer, Swiss Mountain Dog, West Highland White Terrier.

The median age at the time of diagnosis was 4.00 years (0.35 to 11.1 years). Of the 35 dogs nine dogs were euthanized after a median of 790 days (185 to 2,376 days) after the first MRI. 19/35 dogs were still alive at the time of data collection (2023, 2024), while no further information was available for 7/35 dogs.

### 3.1 Findings in follow-up examinations

**Figure 1:**
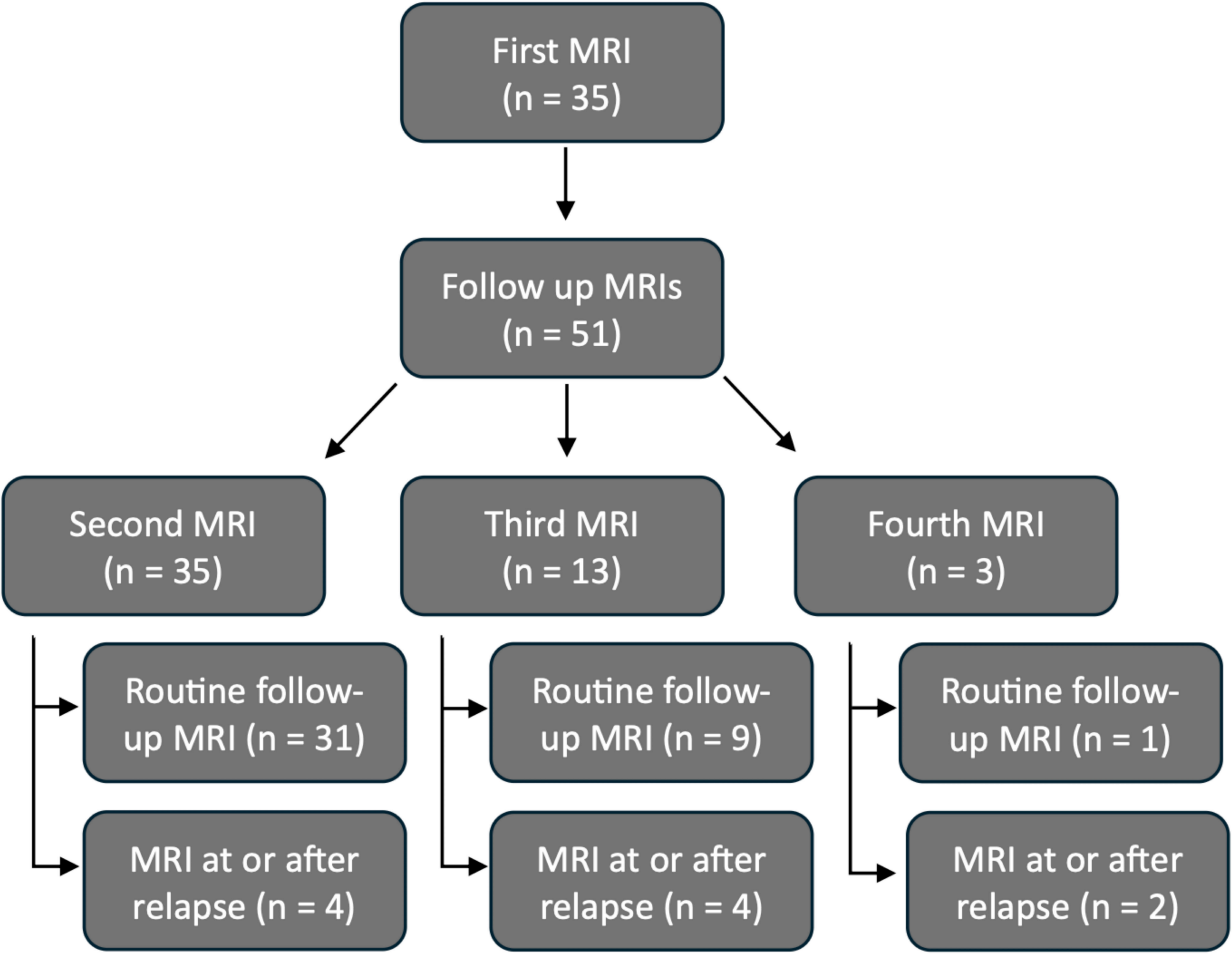
Flow chart showing the number of the magnet resonance imaging (MRI) examinations performed (*n* = 86). The initial MRI examination (*n* = 35) and follow-up examinations (*n* = 51) were shown. The follow-up MRIs are further subdivided according to the number of MRI examinations performed (second to fourth MRI). These were further subdivided into routine follow-up MRIs examinations and MRI examinations due to a relapse or after a relapse.

A total of 86 examinations were analyzed, including MRI, CSF, clinical signs (expressed as NDS), and medication. Not all data and MRI sequences were available for all dogs at every time point. The first MRI was performed at diagnosis in all 35 dogs. The remaining 51 MRIs were follow-up examinations. By inclusion criteria, all dogs (35/35) underwent at least two MRI examinations; 13/35 had three, and 3/35 had four examinations (Figure 1).

**Figure 2:**
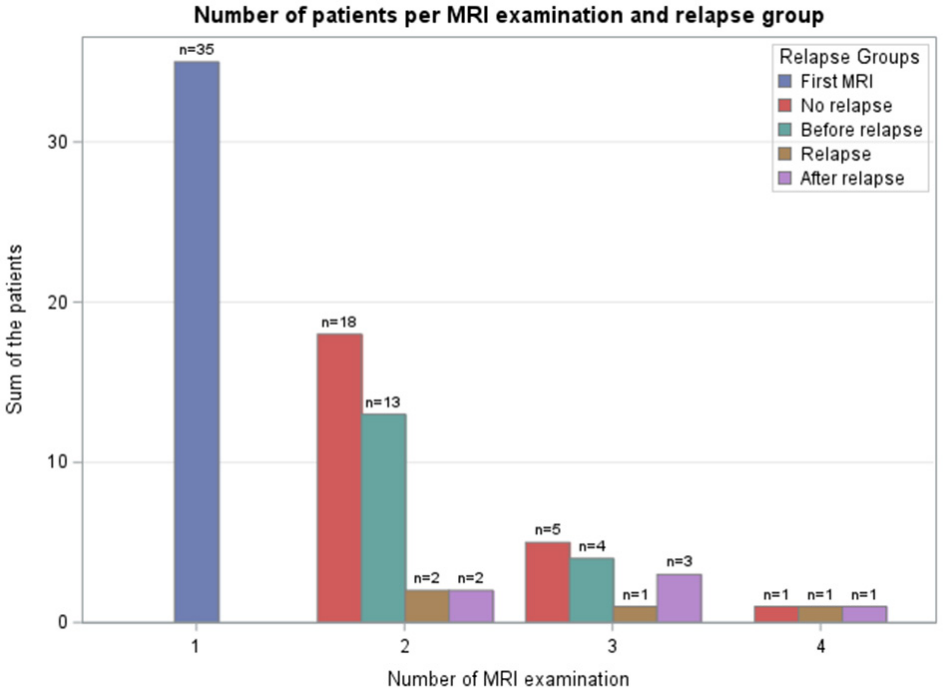
Bar chart for number of patients in relation to the number of magnetic resonance imaging (MRI) examination performed and differentiated by clinical relapse groups. Definition of relapse: in a follow-up examination a higher neurodisabilty scale (NDS) in one or more points, compared to a previous examination.

The second MRI was performed at or after relapse in 4/35 cases, the third in 4/13 cases, and the fourth in 2/3 cases. The remaining 41/51 follow-up MRIs were routine controls without signs of relapse at the time of imaging (Figure 2).

### 3.2 Comparison of findings according to MRI examination timepoint

For detailed results, see Supplementary Table 1. After the first MRI, each dog received a median dose of prednisolone of 1.04 mg/kg/day (0.84–3.28 mg/kg/day). At the second MRI examination, the median prednisolone dose was 0.71 mg/kg/day (0.00–1.25 mg/kg/day). At later MRI examinations, when available, the median prednisolone dose was 0.49 mg/kg/day (0.00–2.66 mg/kg/day) at the time of the third MRI, and 0.78 mg/kg/day (0.00 −1.11 mg/kg/day) at the time of the fourth MRI. During the disease course, 29 dogs received one or multiple add-on immunomodulatory drugs (*n* = 19/29 cytarabine arabinose s.c., *n* = 18/29 ciclosporin p.o., *n* = 2/29 mofetil mycophenolate p.o.).

**Figure 3:**
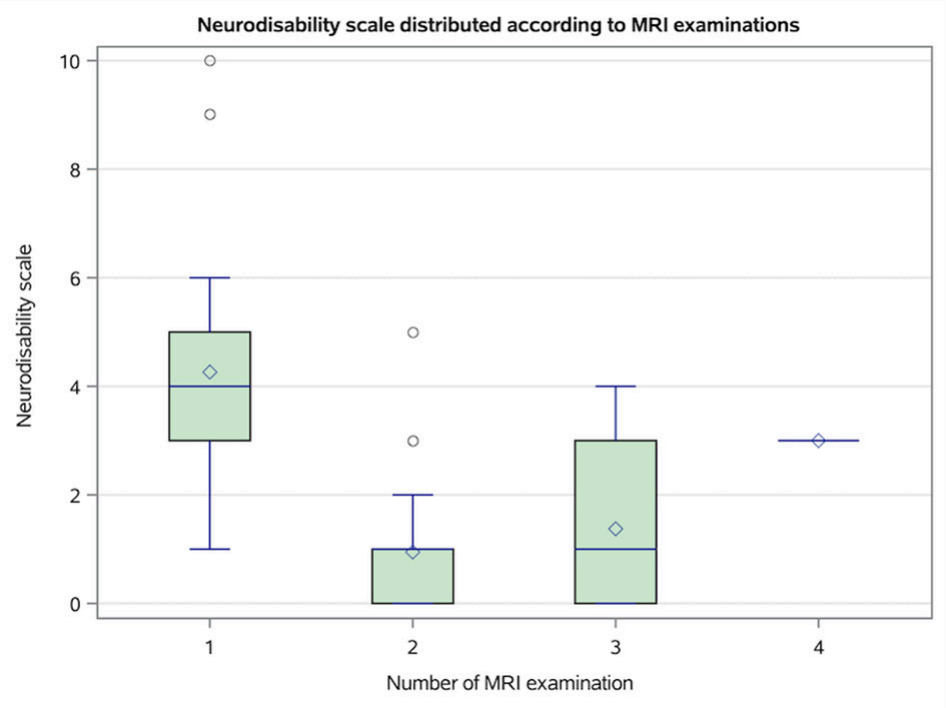
Box plot shows the distribution of the neurodisability scale (NDS) by the number of magnetic resonance imaging (MRI) sequences. Boxes show the interquartile range (25th to 75th percentiles) with a line at the median; diamond inside boxes indicate the arithmetic mean; whiskers show the most extreme values within or 1.5 × interquartile range, and observation beyond (outliners) are plotted as points.

The neurodisability scale (NDS) differed significantly between MRI examinations timepoints (*p* < 0.0001). NDS at the first MRI was significantly higher than that at the second (*p* < 0.0001) and third MRI (*p* = 0.0393), but did not differ significantly the fourth MRI examination (*p* > 0.05; Figure 3).

**Figure 4:**
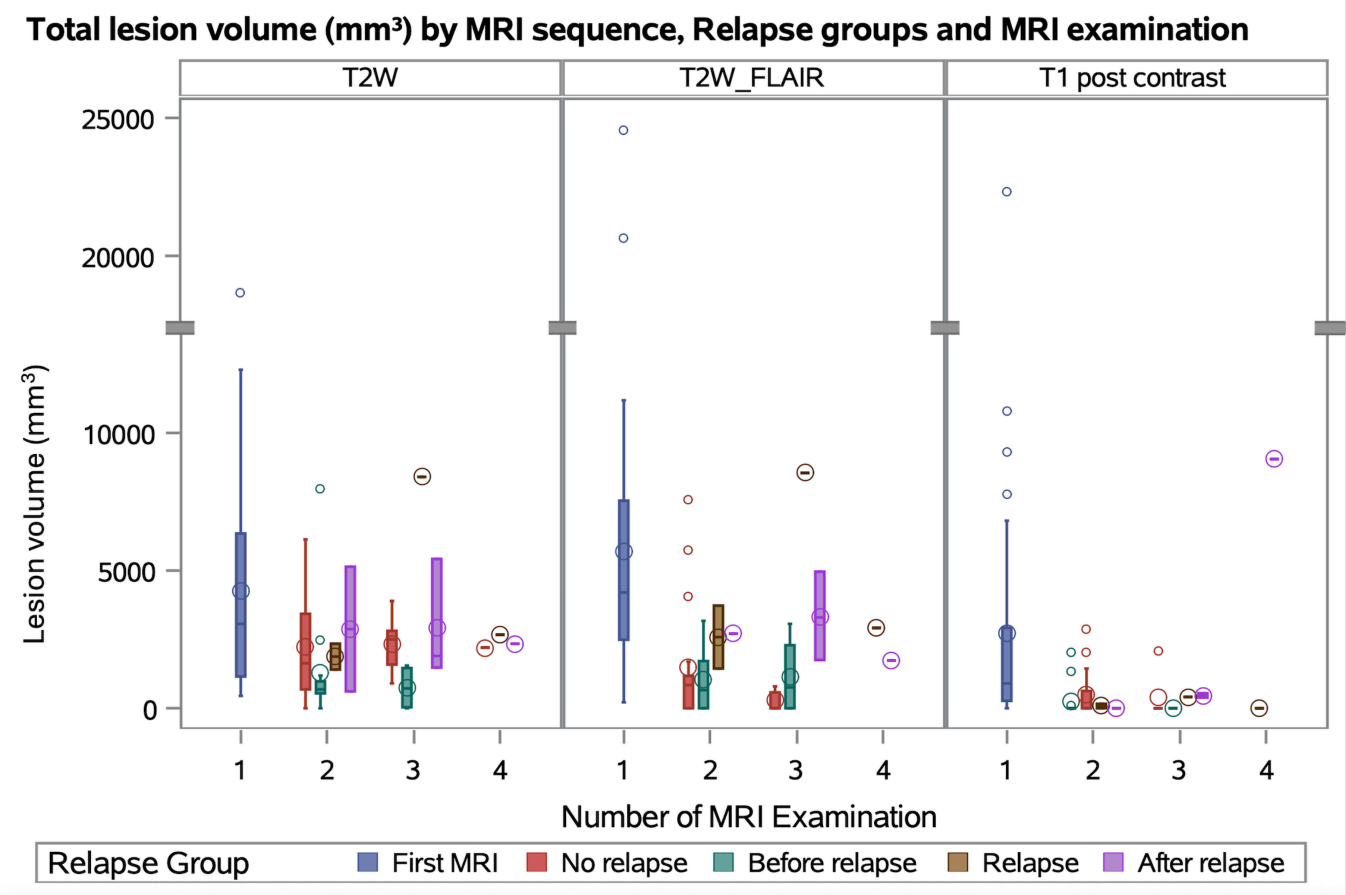
Box plot shows the distribution of lesion volume in mm^3^ by magnetic resonance imaging (MRI) sequences, Number of MRI and relapse groups. Definition of relapse: in a follow-up examination a higher neurodisability scale (NDS) in one or more points, compared to a previous examination. T2 weighted (T2w): lesion volume in first MRI to before relapse were significant different (*p* = 0.0023). FLAIR: lesion volume in first MRI to before relapse (*p* = 0.0003) and no relapse (*p* = < 0.0001) were significant different. T1 post contrast: lesion volume in first MRI to before relapse (*p* = 0.0005) and no relapse (*p* = 0.0017) were significant different. Boxes show the interquartile range (25th to 75th percentiles) with a line at the median; circle inside boxes indicate the arithmetic mean; whiskers show the most extreme values within or 1.5 × interquartile range, and observation beyond (outliners) are plotted as points.

Total and relative lesion volume in T2w, FLAIR and contrast enhancement were significantly different between the MRI examinations (all *p* < 0.02). Pairwise comparisons demonstrated significant differences between the first and the second MRI (*p* < 0.02) and in FLAIR and contrast enhancement between the first and third MRI among dogs with three MRI examinations available (*p* < 0.05), but not in comparison with the fourth MRI (*p* > 0.05; Figure 4).

While the number of lesions in T2w was not significantly different between the MRI examinations (*p* > 0.05), the number of lesions in FLAIR and contrast enhancement was significantly different between MRI examinations (*p* < 0.01), with the first MRI and the fourth MRI showing the highest lesion numbers.

The MRI examinations differed significantly for the numbers of new lesions in FLAIR, T2w, and contrast enhancement (*p* < 0.0001). In T2w or FLAIR, 10/35 and 6/32 dogs developed new lesions at the second MRI examination compared to the previous MRI. 6/13 dogs developed new lesions on their third MRI examination comapred to the previous. 2/3 and 1/2 dogs developed new lesions on their fourth MRI examination compared to the previous MRI. In the second MRI no new T1 contrast enhancing lesions were found (*n* = 0/32), while in the third 3/11 and in the fourth MRI 1/2 dogs showed new contrast enhancing lesions compared to the previous.

CSF findings, namely WBC count, protein, albumin, and Qalb, were significantly different between examinations (all *p* < 0.002) and improved after the initial examination. The proportions of lymphocytes and macrophages did not differ significantly between examinations (*p* > 0.05). In contrast, the proportion of neutrophilic granulocytes differed significantly between MRI time points (*p* = 0.0274) and decreased after the first MRI and was again higher at the time of the fourth MRI examination.

### 3.3 Comparison of findings according to the reason for MRI examination

For detailed results, see Supplementary Table 2. The initial MRI was performed at diagnosis of MUO (*n* = 35/86) on day 0. Routine follow-up examinations to monitor therapy in dogs without relapse were conducted in 41/86 cases at a median of 120 days (65–443 days) after diagnosis. In 10/86 cases with relapse, MRI was performed either at the time of relapse (*n* = 4) a median of 312.5 days (183–483 days) after the initial diagnosis, or after relapse (*n* = 6) at a median of 1,123.5 days (329–1,528 days) after the initial diagnosis.

**Figure 5:**
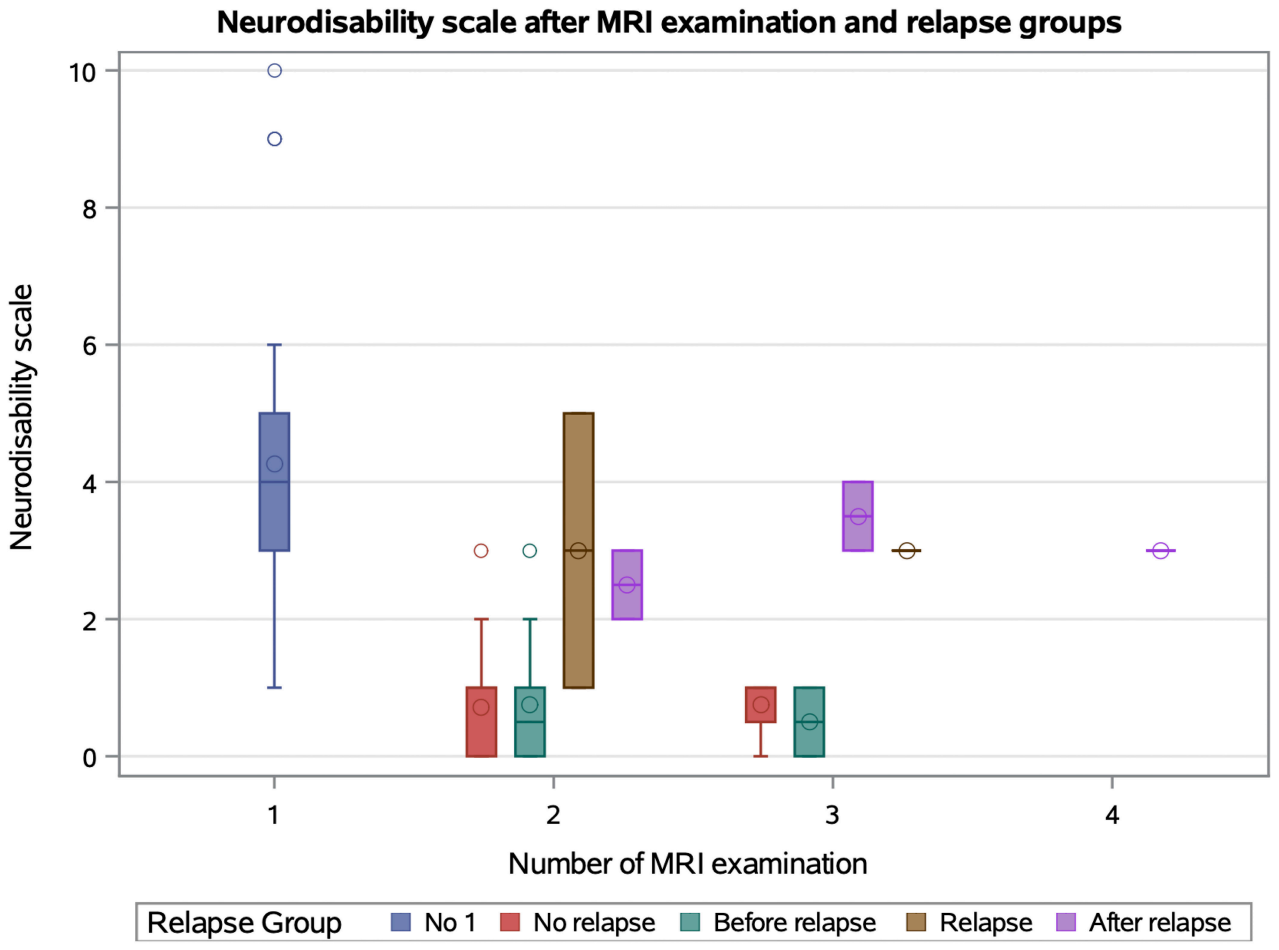
Box plot shows the distribution of the neurodisability scale (NDS) by the relapse groups and the number of magnetic resonance imaging (MRI). Definition of relapse: in a follow-up examination a higher neurodisability scale (NDS) in one or more points, compared to a previous examination. Boxes show the interquartile range (25th to 75th percentiles) with a line at the median; circle inside boxes indicate the arithmetic mean; whiskers show the most extreme values within or 1.5 × interquartile range, and observation beyond (outliners) are plotted as points.

NDS differed significantly between MRI examinations performed for clinical reasons (*p* < 0.0001), being lower at routine follow-up than at diagnosis (*p* < 0.0001) and higher after relapse compared to routine follow- up (*p* = 0.0019; Figure 5).

Total and relative lesion volumes in T2w, FLAIR, and contrast-enhanced sequences differed significantly between MRI examinations (all *p* < 0.01). Pairwise comparisons showed significant differences between routine follow-up MRIs and the initial MRI at MUO diagnosis (all *p* < 0.005), as well as between MRIs performed at or after relapse and the initial MRI (all *p* < 0.05).

**Figure 6:**
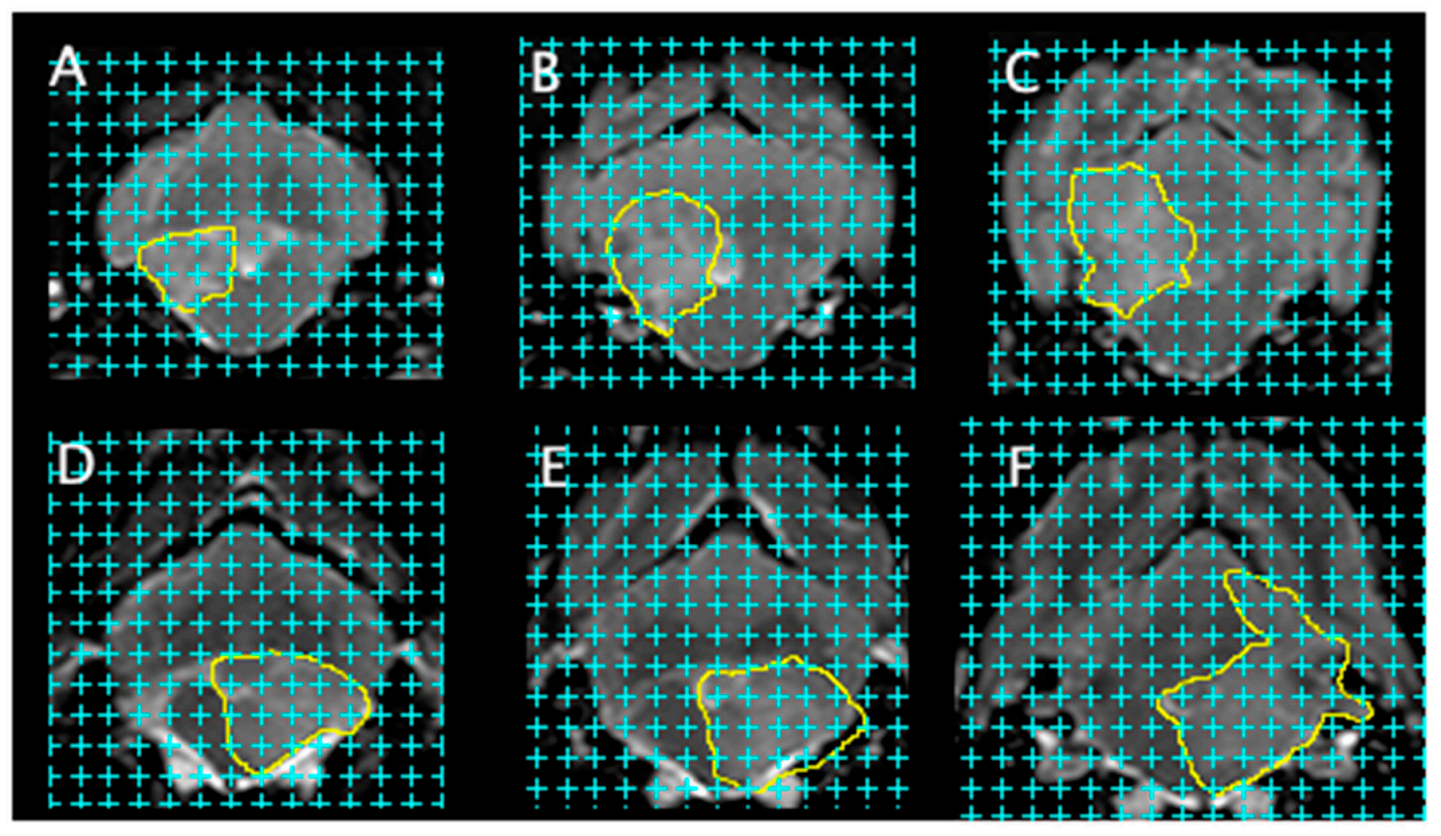
Transverse T2 weighted (T2w) brain magnet resonance images of two different dogs with meningoencephalitis of unknown origin (MUO). These images illustrating lesion appearance and the lesion volume assessment workflow. Lesions were manually outline (yellow line) and a 12 mm^2^ point-counting grid was applied with (cyan crosses) for lesion estimation (Cavalieri method). **(A–C)** and **(D–F)** shows example examinations from two dogs with MUO.

Representative examples illustrating lesion appearance and the relative lesion volume measurements are shown in Figure 6.

**Figure 7:**
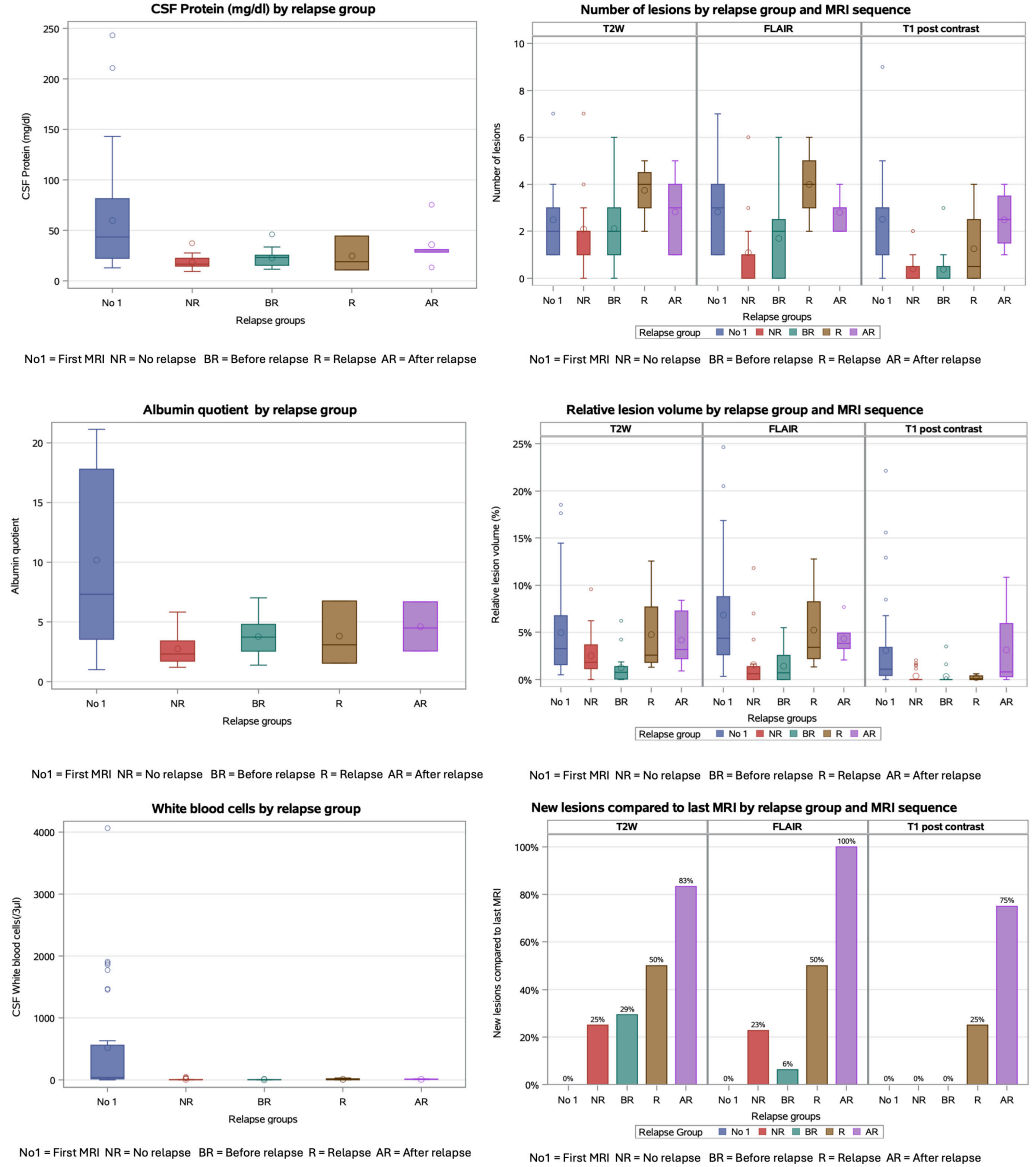
Box plots and bar chart of certain values for cerebrospinal fluid (CSF) and magnetic resonance imaging (MRI) for the relapse groups. The MRI values are additionally divided into the MRI Sequences. Definition of relapse: in a follow-up examination a higher neurodisability scale (NDS) in one or more points, compared to a previous examination. Boxes show the interquartile range (25th−75th percentiles) with a line at the median; circle inside boxes indicate the arithmetic mean; whiskers show the most extreme values within or 1.5x interquartile range, and observation beyond (outliners) are plotted as points. T2W, T2-weighted; FLAIR, fluid attenuated inversion recovery; T1 post contrast: T1-weigthed post contrast.

The number of lesions in T2w images did not differ significantly between MRI examinations (*p* > 0.05). In contrast, lesion numbers in FLAIR and contrast-enhanced sequences differed significantly between examinations (*p* < 0.0001). The initial MRI had a significantly higher lesion numbers than routine follow-up MRIs (median: FLAIR 3 vs. 1 lesion; T1w post contrast 3 vs. 0 lesions, all *p* < 0.001). Routine follow-up MRIs also differed significantly in the number of lesions in FLAIR images at time of relapse (median 1 vs. 4) and in post contrast images after relapse (median: 0 vs. 2.5; both < 0.05; Figure 7).

In T2w and FLAIR sequences, dogs developed new lesions at the routine follow-up MRIs compared to the previous MRI in 11/41 and 6/38 MRI examinations, respectively. At relapse, new lesions were observed in both T2w and FLAIR sequences in 2/4 dogs. After a relapse, new lesions were identified in 5/6 dogs in T2w and 5/5 dogs in FLAIR (*p* < 0.0001).

No new T1 contrast-enhancing lesions were detected during routine follow-up (0/37 dogs). Conversely, at or after relapse, 1/4 and 3/4 dogs, respectively, showed new contrast-enhancing lesions compared to the previous MRI (*p* < 0.0001; Figure 7).

**Figure 8:**
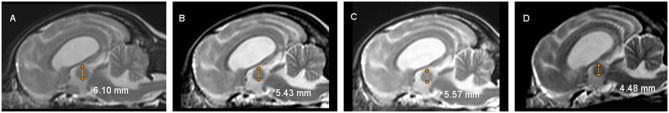
Sagittal magnetic resonance imaging (MRI) T2w brain image of a 5 years old neutered female Maltese dog with measuring of the interthalamic adhesion high. **(A)** Interthalamic adhesion (6.10 mm) height at time of diagnosis. **(B, C)** Interthalamic adhesion (5.43/5.57 mm) height in a routine follow up MRI. **(D)** interthalamic adhesion (4.48 mm) height after a relapse.

Brain volume or interthalamic adhesion height as signs of brain atrophy, did not differ significantly when examinations were grouped by MRI time point (*p* > 0.05). Both parameters were positively correlated with body weight (*r* = 0.30, *p* = 0.0081 and *r* = 0.90, *p* < 0.0001). However, when examinations were grouped by relapse status, interthalamic adhesion height differed significantly between groups (*p* = 0.0255) and was smaller after relapse than at diagnosis (*p* = 0.0397; Figure 8).

CSF findings, including WBC count, protein, albumin, and Qalb, differed significantly between examinations (all *p* < 0.05) and were mostly different between the diagnostic examination for MUO and routine follow-up examinations (all *p* < 0.01; Figure 7). The percentage of lymphocytes did not differ significantly between groups (*p* > 0.05). In contrast, the proportions of neutrophilic granulocytes and macrophages differed significantly between groups (all *p* < 0.05), although pairwise comparisons between individual examinations revealed no significant differences (*p* > 0.05).

### 3.4 Predictors of relapse

For detailed results, see Supplementary Table 4. Relapse occurred at a median of 351 days (83–1,527 days) after initial MRI examination in 17/35 dogs.

At diagnosis, neither examination results nor signalment differed significantly between dogs that relapsed and those without clinical relapse (all *p* > 0.05).

A total of 41 routine follow-up MRI examinations were included in the analysis. Of these, 17/41 examinations (from 13 dogs) were followed by relapse, while 24/41 examinations (from 18 dogs) were not followed by any relapse.

In routine follow-up examinations, absolute and relative T2w lesion volumes were significantly different between MRIs followed by relapse and those not followed by relapse (both *p* < 0.05), with lower median lesion volumes observed in MRIs followed by relapse.

A morphologically normal brain MRI was an uncommon finding in both groups: only 2/17 MRIs were normal in dogs with subsequent relapse, and 1/24 MRIs in dogs without relapse were classified as normal, with no significant difference between groups (*p* > 0.05). No other MRI finding differed significantly between routine follow-up scans with subsequent relapse and those without.

**Figure 9:**
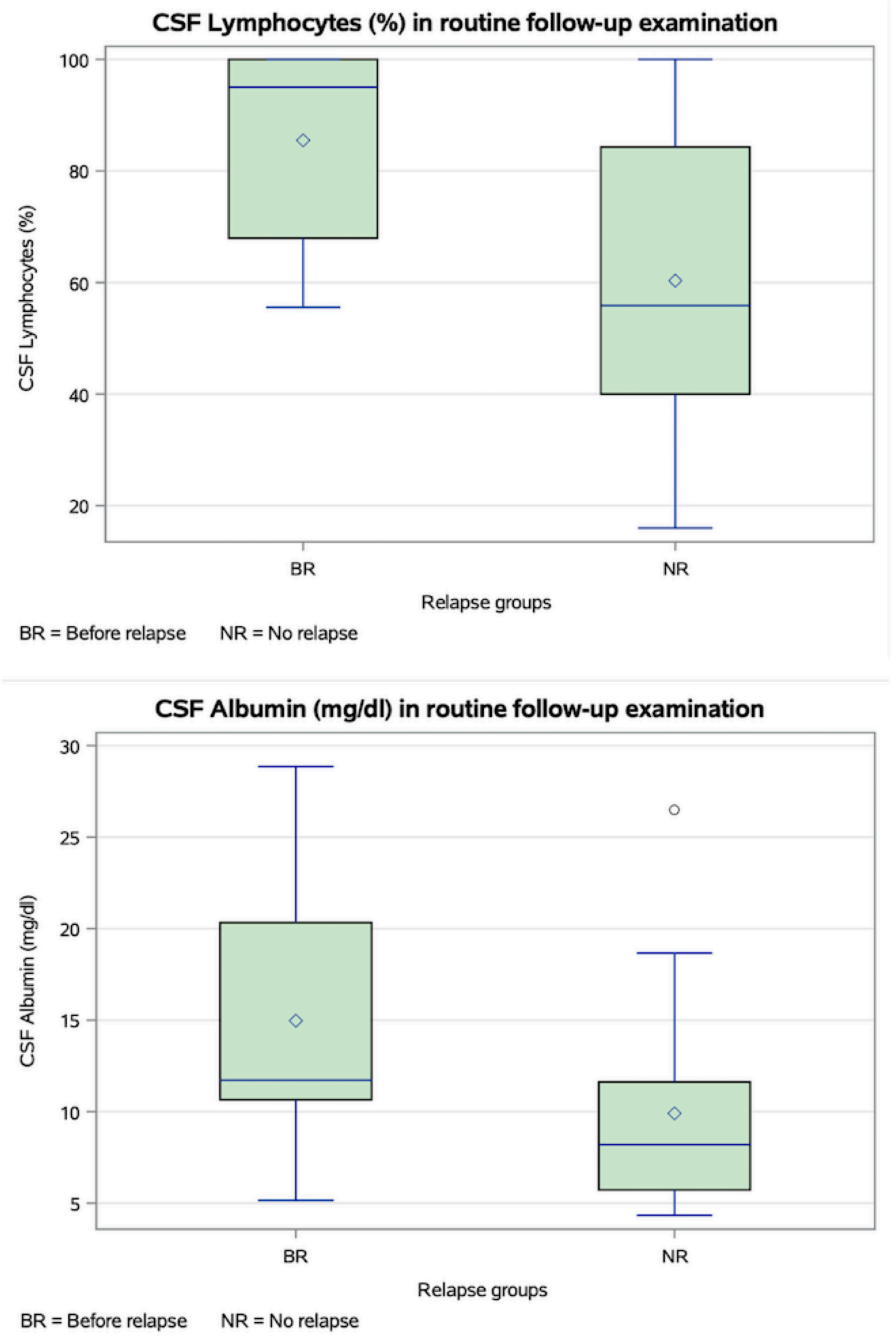
Box plots of cerebrospinal fluid (CSF) albumin (mg/dl) and lymphocytes (%) measured in routine follow-up examination in dogs with subsequent relapse and without a relapse. CSF lymphocytes (*p* = 0.0329) and CSF albumin (*p* = 0.0222) were significantly higher in dogs with a subsequent relapse (BR) compared to those without relapse. Definition of relapse: in a follow-up examination a higher neurodisability scale (NDS) in one or more points, compared to a previous examination. Boxes show the interquartile range (25th to 75th percentiles) with a line at the median; diamont inside boxes indicate the arithmetic mean; whiskers show the most extreme values within or 1.5 × interquartile range, and observation beyond (outliners) are plotted as points.

CSF albumin was significantly higher in routine follow-up examinations followed by relapse compared with those not followed by relapse (*p* = 0.0222), whereas QAlb did not differ significantly between groups (*p* > 0.05). CSF albumin levels were positively associated with QAlb (*p* < 0.0001). The proportion of CSF lymphocytes were also significantly different between these groups (*p* = 0.0329; Figure 9).

Groups did not differ significantly regarding treatment. Only the decrease in medication after the MRI examination was significantly different (*p* = 0.0496), with a more frequent reduction in medication in dogs without relapse.

A binary logistic regression model identified predictors of relapse at routine follow-up examinations. Higher CSF albumin levels (*p* = 0.0413) and a higher proportion of CSF lymphocytes (*p* = 0.0288) were associated with an increased risk of relapse. In contrast, a reduction in medication after MRI was associated with a decreased risk of relapse (*p* = 0.0476; Table 1).

**Table 1:** Logistic regression of predictors of relapse.

**Variable**		**Odds ratio**	***p*-value**	**95% Confidence interval**	**Coefficient (β)**
CSF	WBC (cells/3 μl)	0.936	0.2541	0.835–1.049	−0.0665
	Total protein (mg/dl)	1.075	0.1466	0.975–1.185	0.0721
	Albumin (mg/dl)	**1.151**	**0.0413**	**1.006–1.318**	**0.1409**
	Qalb (mg/dl)	1.570	0.0958	0.923–2.668	0.4508
	Neutrophilic granulocytes (%)	0.966	0.1744	0.918–1.016	−0.0351
	Lymphocytes (%)	**1.045**	**0.0288**	**1.005–1.087**	**0.0438**
	Large monocytes (%)	0.977	0.2538	0.938–1.017	−0.0237
Medication	Increased medication	3.0667	0.5598	0.255–36.878	
	Decreased medication	**0.2339**	**0.0476**	**0.0594–0.9207**	
	Add on medication	1.3382	0.7364	0.802–3.042	
	Dose prednisone	7.495	0.0762	0.809–69.450	2.0143

Logistic regression of possible predictors in routine examination [at the time of magnetic resonance imaging (MRI) examination] associated with a risk of a relapse between group no relapse and before relapse.

Significant level for p < 0.05. Bold values indicate statistical significance (*p* < 0.05).

**Figure 10:**
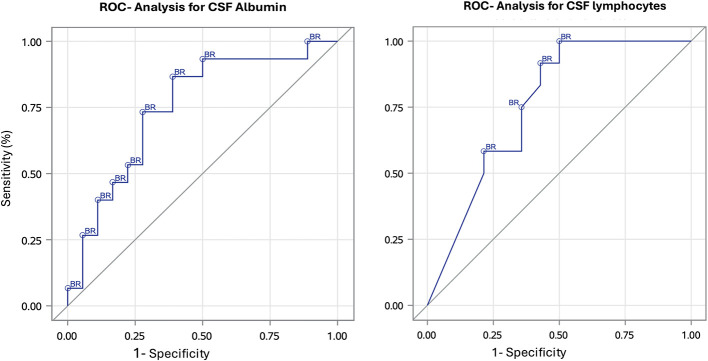
Receiver operating characteristic (ROS) curves for cerebrospinal fluid (CSF) albumin (mg/dl) and lymphocytes (%) measured at routine follow-up. Optimal threshold (Youden index): CSF albumin 9.64 mg/dl (sensitivity of 86.7% and a specificity of 61.1%); CSF lymphocytes 74.0% (sensitivity of 66.7% and a specificity of 64.3%). BR, before relapse [Examinations before deterioration of the neurodisability scale (NDS)].

ROC analysis with Youden index optimization yielded a threshold value of 9.64 mg/dl for CSF albumin (sensitivity of 86.7% and a specificity of 61.1%) and 74.0% for CSF lymphocytes (sensitivity of 66.7% and a specificity of 64.3%; Figure 10).

### 3.5 Association of clinical signs with MRI findings

NDS was significantly associated with total and relative lesion volume in T2w (total volume: *p* = 0.0027; relative volume: *p* = 0.0027) and FLAIR (total volume: *p* = 0.0034; relative volume: *p* = 0.0005) and with the number of T1 contrast enhancing lesions (*p* = 0.0006; Table 2).

**Table 2:** Factors associated with neurodisabilty scale (NDS).

**Variable**	**Coefficient (β)**	**Standard Error (SE)**	***p*-value**
**Total lesion volume, absolute**			
T2w	−0.00061	0.000186	**0.0027**
FLAIR	−0.00048	0.000148	**0.0034**
T1w contrast enhancement	−0.00040	0.000245	0.1131
**Total lesion volume, relative (%)**			
T2w	0.5921	0.1552	**0.0006**
FLAIR	0.4897	0.1227	**0.0005**
T1w contrast enhancement	0.4279	0.2150	0.0558
**Number of lesions**			
T2w	0.3926	0.1143	**0.0018**
FLAIR	0.6961	0.1220	0.5730
T1w contrast enhancement	0.6592	0.1725	**0.0006**
**Lesion in cerebellum**			
T2w	−0.6416	0.6809	0.3473
FLAIR	−1.3588	0.4924	**0.0064**
T1w contrast enhancement	−1.3105	0.5580	**0.0203**
**Lesion in brainstem**			
T2w	−1.1229	0.4395	**0.0114**
FLAIR	−0.9117	0.3922	**0.0213**
T1w contrast enhancement	−2.6295	0.5461	**< 0.0001**
**Lesion in forebrain**			
T2w	−0.2760	0.4724	0.5597
FLAIR	−0.8862	0.4239	**0.0381**
T1w contrast enhancement	−1.6403	0.5202	**0.0020**
**Lesion in piriform lobe**			
T2w	−0.1865	0.4497	0.6788
FLAIR	0.5029	0.4701	0.2863
T1w contrast enhancement	−3.2682	1.2747	**0.0114**
**Lesion meninges**			
T2w	−0.1835	0.3612	0.6121
FLAIR	−1.2890	0.4620	**0.0059**
T1w contrast enhancement	0.5724	0.5499	0.2998
Loss of sulci (T2w)	−1.7162	0.3700	**< 0.0001**

Negative coefficient for binary nominal variables (reference: lesion present) indicates that dogs without a lesion in this brain area have a decreased NDS.

Positive coefficient for continuous variables indicates that dogs with higher values are associated with an increased NDS.

Significant level for p < 0.05 is labeled in bold.

Higher NDS was associated with the presence of lesions in the brainstem in T2w (*p* = 0.0114) or FLAIR (*p* = 0.0213), visible lesions in the meninges (*p* = 0.0059), forebrain (*p* = 0.0381), and cerebellum (*p* = 0.0064) in FLAIR, and loss of sulci (*p* < 0.0001). Higher NDS was also associated with contrast enhancement of the forebrain (*p* = 0.0020), the piriform lobe (*p* = 0.0114), the brainstem (*p* < 0.0001), and the cerebellum (*p* = 0.0203; Table 2).

The risk of epileptic seizures increased with a higher total lesion volume (*p* = 0.0485) and a higher relative lesion volume (*p* = 0.0170) in T2w. Lesions in the piriform lobe (T2w: *p* = 0.0001; FLAIR: *p* < 0.0001) and in the frontal lobe (T2w: *p* = 0.0102; FLAIR: *p* = 0.0218), and a higher relative volume of contrast enhancement (*p* = 0.0309) or contrast enhancement in the frontal lobe (*p* = 0.0151; Table 3).

**Table 3:** Factors associated with seizures.

**Variable**	**Odds ratio**	***p*-value**	**95% Confidence interval**	**Coefficient (β)**
**Total lesion volume, absolute**				
T2w	1.000	**0.0485**	1.000–1.000	0.000167
FLAIR	1.000	0.0997	1.000–1.000	0.000100
T1w contrast enhancement	1.000	0.0618	1.000–1.000	0.000173
**Total lesion volume, relative (%)**				
T2w	1.194	**0.0170**	1.032–1.381	0.1773
FLAIR	1.108	0.0525	0.999–1.230	0.1028
T1w contrast enhancement	1.184	**0.0309**	1.016–1.318	0.0309
Interthalamic adhesion size (mm)	0.431	**0.0130**	0.222–0.837	0.0130
Brain volume (mm^3^)	1.000	0.2367	1.000–1.000	−0.00002
**Lesion in piriform lobe**				
T2w	0.113	**0.0001**	0.037–0.342	−2.1833
FLAIR	0.091	**< 0.0001**	0.030–0.278	−2.3998
T1w contrast enhancement	0.955	0.9712	0.081–1.327	−0.0456
**Lesion in frontal lobe**				
T2w	0.209	**0.0102**	0.064–0.690	−1.5632
FLAIR	0.238	**0.0218**	0.070–0.811	−1.4352
T1w contrast enhancement	0.185	**0.0151**	0.047–0.721	−1.6896

Binar logistic regression based on the estimation of the probability of a seizure occurring (seizure = “Yes”).

Negative regression coefficient in nominal variables (reference category: lesion present) indicates that dogs without a lesion have a decreased risk of seizure.

Positive regression coefficient in continuous variables indicates that increased values are associated with an increased risk of seizure.

Significant level for p < 0.05 is labeled in bold.

## 4 Discussion

This study investigated longitudinal changes in MRI findings, neurological status (NDS), and CSF parameters in dogs with MUO undergoing immunosuppressive therapy. Follow-up evaluations, including MRI, neurological examination, and CSF analysis, were compared between routine follow-up visits and examinations performed at or after clinical relapse to identify potential predictors of relapse.

In our cohort, newly appearing lesions on T2w and FLAIR images, as well as new contrast enhancing lesions occurred significantly more often in examinations performed after a relapse than during follow-up MRI examinations (*p* ≤ 0.0029), supporting these MRI changes as markers of current disease activity and potentially reduced treatment response. Newly occurring lesions also indicated disease progression. Beyond imaging, CSF albumin and lymphocytes emerged as the most clinically relevant predictors of a subsequent relapse.

### 4.1 Findings at follow up examinations

Routine follow-up MRI examinations (predominantly MRI examination two and three, performed in the absence of clincal relapse) showed a continuous reduction in lesion volume across T2w, FLAIR, and T1 post-contrast sequences during immunosuppressive therapy. In contrast, lesion counts did not decrease consistently over time, suggesting that lesion volume is a more sensitive marker of treatment response and inflammatory activity than lesion number. This supports the use of volumetric assessment in follow-up imaging to better evaluate disease regression.

CSF analysis supported the imaging and clinical findings by demonstrating a reduction in inflammatory activity during treatment. Routine follow-up examinations showed lower WBC counts, protein, albumin, and QAlb compared with baseline, consistent with therapeutic suppression of CNS inflammation.

Parallel improvement in neurological status, reflected by lower NDS, mirrored the reduction in lesion volume. This association between lesion load and clinical course accords with previous findings, where higher lesion volume at the time of diagnosis was linked to higher NDS (8).

#### 4.1.1 Imaging features associated with relapse

In dogs with a fourth MRI examination, lesion volumes increased again across all sequences, reversing the gradual reduction observed during earlier follow-ups. This was most likely because MRI was predominantly performed at or after clinical relapse in the population examined at a fourth time point, when renewed inflammatory activity was present. Active relapse was reflected by increased lesion burden, the reappearance of contrast enhancement, and higher NDS values, indicating a parallel deterioration in clinical status. While lesion counts in T2w images did not differ significantly across examinations, lesion numbers in FLAIR and contrast-enhanced sequences were significantly higher, with the MRI examination at a fourth time point and the initial MRI showing the greatest counts. These findings underscore that both lesion volume and specific sequence-based lesion counts, alongside clinical scoring, provide complementary information to confirm relapse and guide treatment adjustments.

This is more clearly seen when MRI examinations are categorized according to the reason for imaging (initial diagnosis, routine follow-up, or at/after relapse). Under immunosuppressive therapy, routine follow-up MRIs showed a reduction in both absolute and relative lesion volumes, accompanied by clinical improvement and reduced relapse activity. In contrast, MRIs performed at or after relapse revealed increased lesion volumes, particularly in dogs examined after relapse, which paralleled worsening NDS scores. The number of lesions and the number of new lesions were also higher at relapse and after relapse compared with the preceding MRI, with the most pronounced increases seen in new contrast-enhancing lesions. These findings indicate that relapse is associated with renewed active inflammation, likely due to reactivation of existing lesions and/or the formation of new lesions. This is consistent with findings by Soulé et al. (20). In their study, dogs experiencing relapse after 6 months had more lesions overall, both newly developed and pre-existing lesions that had enlarged, and showed more frequent contrast enhancement. The authors proposed that this pattern reflects an ongoing or reactive inflammatory process and noted that individual lesions may respond differently to immunosuppressive therapy (20). Similarly, Gonçalves et al. (16) reported that, while the mere presence of contrast-enhancing lesions was not associated with relapse, the risk of relapse increased with a higher number of such lesions.

#### 4.1.2 Brain atrophy during follow-up and after relapse

During treatment, no significant changes consistent with brain atrophy were detected across examinations time points. However, when grouped by relapse status, brain volume decreased slightly, as indicated by a reduction in interthalamic adhesion height. This may indicate that the brain is swollen at diagnosis or that atrophy develops following relapse. Interpretation is limited, as interthalamic adhesion height is positively correlated with both brain volume and body weight. Previous studies have shown that interthalamic adhesion height, alone or in combination with other parameters, can serve as a marker for brain atrophy in dogs with cognitive dysfunction (17, 21). In addition, a recent study investigating brain atrophy in MUO used various volumetric measures, including interthalamic adhesion thickness-to-brain height ratio, and found that atrophy occurs in dogs with MUO, with more severe atrophy associated with poorer prognosis (13). These findings suggest that interthalamic adhesion height may also serve as a useful marker for brain atrophy in dogs with MUO.

### 4.2 Predictors of relapse

#### 4.2.1 MRI derived predictors of relapse

Normal MRI during follow-up examination was rare in this study. Only three follow-up MRIs were normal, two of which were followed by subsequent relapse. While numbers are small, this observation suggests that a normal follow-up MRI may not reliably indicate relapse free disease. In contrast, another study demonstrated that dogs with a normal MRI at time of diagnosis had better long-term prognosis than dogs with abnormal MRIs (22). These results suggest that normal MRI at time of diagnosis may indicate a good long-term prognosis, whereas a normal MRI during follow-up should be interpreted cautiously and does not imply relapse freedom.

Absolute and relative lesion volumes on T2-weighted images were significantly higher in dogs that did not experience a subsequent relapse compared to those that did. One possible explanation is that the visibly larger lesions led veterinarians to be more cautious in tapering immunosuppressive therapy, which may have helped prevent relapse. No other MRI findings differed between dogs that did not experience a subsequent relapse compared to those that did at the timepoint of routine follow-up examinations.

New lesions on routine follow-up MRIs in T2w or FLAIR sequences did not necessarily predict future relapse. Dogs without relapse even showed an increase in new lesions, particularly in FLAIR sequences (5/22 dogs compared to 1/16 dogs), although this difference was not statistically significant. None of the dogs undergoing routine follow-up MRI developed new contrast-enhancing lesions. It is possible that the detection of new lesions during follow-up imaging influenced clinical decision-making, leading veterinarians to prolong prednisone treatment or avoid therapy reduction. Such an effect could have reduced the risk of relapse in these cases, although this cannot be confirmed statistically in the present study.

#### 4.2.2 CSF derived predictors of relapse

CSF analysis at routine follow-up examinations revealed significantly higher albumin concentrations in dogs with subsequent relapse (median 11.72 mg/dl) compared to those without relapse (median 8.19 mg/dl). In contrast, QAlb—a marker of blood–brain barrier (BBB) integrity—did not differ significantly between groups, although it was correlated with CSF albumin levels. This suggests that total albumin concentration, rather than BBB disruption alone, may play a role in the pathophysiology of MUO or MUO relapse. Experimental evidence indicates that albumin can activate glial cells via the transforming growth factor beta receptor (TGFβR) and the canonical TGFβR–smad signaling pathway, increasing phosphorylation of smad2 and smad3 and promoting nuclear translocation of smad4 (23, 24). Albumin also induces the pro-inflammatory cytokine IL-1β in astrocytes and microglia through TGFβR-dependent but smad-independent mechanisms (24). These effects, together with TGFβR-mediated cytotoxicity and other signaling changes, support a role for albumin in perpetuating CNS inflammation (24). In our logistic regression model, each 1 mg/dl increase in CSF albumin was associated with a 15% higher probability of relapse and might therefore be a more valuable prognostic marker than MRI. The ROC analysis identified a CSF albumin concentration above 9.64 mg/dl as being associated with relapse, with a sensitivity of 86.7% and a specificity of 61.1%. The relatively high sensitivity suggests that this threshold may be useful for identifying dogs at increased risk during routine follow-up, whereas the moderate specificity indicates that false-positive results can occur. Further studies are needed to replicate this cut-off value of 9.64 mg/dl in an independent cohort. Until then, this cut-off could be used as part of an integrated clinical assessment together with MRI findings, neurological status, and treatment history.

Cerebrospinal fluid lymphocyte proportions were also significantly higher at follow-up in dogs with subsequent relapse compared to those without. This may indicate that these dogs still had an active adaptive immune response at the time of CSF sampling or were experiencing a recurrence of neuroinflammatory activity. Supporting this interpretation, our binary logistic regression analysis showed that each one percentage-point increase in CSF lymphocytes was associated with an increased risk of relapse. The ROC analysis revealed a threshold of 74.0% CSF lymphocytes as the value at which the risk of relapse is increased, with a sensitivity of 66.7% and a specificity of 64.3%. The moderate sensitivity and specificity suggest that this threshold may support risk stratification during routine follow-up, but that misclassification can occur when used in isolation. Further validation of this cut-off is required to confirm the robustness. Meanwhile, it should be used as supportive information in combination with other clinical and imaging findings. These findings are consistent with immunohistochemical studies showing a predominance of CD3+ T-lymphocytes in active neuroinflammatory lesions in MUO, particularly in cases of granulomatous meningoencephalitis (25). Conversely, dogs without subsequent relapse may have already entered a reparative phase, characterized by declining lymphocyte counts and a predominance of macrophages or neutrophils, which function in tissue remodeling and repair.

Elevated CSF WBC counts were not predictive of relapse in this study. One possible explanation is that in dogs with high WBC counts, the treating veterinarian may have refrained from tapering medication or may have intensified treatment as a precautionary measure. This is supported by our logistic regression findings, which suggested a reduced relapse risk in dogs whose medication was increased or maintained rather than reduced after follow-up MRI.

Although therapy type did not differ significantly between relapse and non-relapse groups, dogs without relapse more often had medication reduced, likely due to favorable MRI findings. Treatment decisions may have influenced outcomes: new or enlarged lesions often prompted maintaining therapy, whereas marked MRI improvement encouraged tapering. This aligns with our finding that reduction of medication was associated with lower relapse risk.

### 4.3 Association of clinical signs with MRI findings

#### 4.3.1 NDS and MRI findings

This study found that higher absolute or relative lesion volumes in T2w and FLAIR images were associated with worse NDS. Loss of sulci also correlated with higher NDS, likely reflecting acute, diffuse, and severe inflammation with brain swelling, which can impair multiple neurological functions. This accords with previous findings linking loss of sulci to increased mortality (3) and higher NDS at diagnosis to greater relapse risk (16).

Lesions in the brainstem on any MRI sequence were also associated with increased NDS, as many NDS-assessed functions, such as cranial nerve function, consciousness, balance, gait, and proprioception, depend on brainstem integrity. Contrast enhancement in the brainstem, cerebrum, cerebellum, or meninges was significantly linked to higher NDS, supporting the interpretation that these represent acute inflammatory rather than chronic lesions.

Although brain herniation is known to be associated with poor prognosis (3), it was not assessed in our study because none of the dogs showed brain herniation on the first MRI, likely reflecting selection bias, as such dogs may have died before follow-up imaging. This may have resulted in a study population with less severe clinical disease.

#### 4.3.2 Seizure and MRI localization

Seizures were observed primarily in dogs with lesions in the piriform and frontal lobes, regions previously described as epileptogenic centers. MRI studies during seizures have identified abnormalities in the cingulate gyrus, piriform lobe, and hippocampus (26). Another study found that lesions in the temporal lobe (including the piriform lobe), frontal lobe, parietal lobe, and olfactory bulb significantly increased seizure risk in dogs with neoplasia (27). Furthermore, MUO lesions in the hippocampus and the occurrence of acute symptomatic seizures predicted the development of post encephalitic epilepsy (28). Taken together, these data support that, the localization of MUO lesions on MRI reflects the occurrence of seizures and contributes to explaining the observed clinical symptoms.

This study has several limitations that should be considered when interpreting the results. As a retrospective study, there was no standardized design, and the interval between initial diagnosis and follow-up examinations varied. Patients also received different immunosuppressive treatments, which may have influenced disease progression. Lesion volume measurements were performed with a degree of standardization, for example, by counting all crosses within a lesion. However, MRI evaluations were conducted by a single observer, introducing the potential for subjective bias.

The study was based on three Tesla MRI scans from a single institution, which may limit generalizability.

The NDS is validated, but retrospective application is less reliable (1, 16), especially if clinical records lack detailed neurological descriptions; however, well-documented examinations could make NDS use more consistent in such settings. Additional sources of bias may include survival bias, as dogs with severe disease (e.g., brain herniation) may not have survived to undergo follow-up imaging. In addition, the small numbers of dogs that underwent a fourth MRI examination might limit the statistical power of comparisons involving this time point.

Dogs with normal MRI findings on initial diagnostic MRI were not included to reduce diagnostic ambiguity, even though normal MRI findings have been reported in a minority of dogs with MUO (2). Consequently, our findings and proposed cut-off values may not apply to MUO dogs with a normal diagnostic MRI and should be evaluated in future studies. Morphologically normal findings on some routine follow-up MRI examinations in this study likely reflect radiological improvement under treatment and do not contradict the absence of normal MRIs at diagnosis.

Observer bias in lesion identification, differences in MRI sequence quality, and variations in the timing of follow-up imaging relative to relapse onset could also have influenced results.

## 5 Conclusion

During follow-up examination CSF albumin and lymphocytes were the strongest predictors of an impending relapse. However, Imaging remains essential to identifying active lesions, especially the appearance of new contrast- enhancing lesions should be interpreted as a marker of active inflammation and ongoing relapse. These results support cautious reduction of immunosuppressive medication and only after objective stabilization has been confirmed by both imaging and CSF findings.

## Data Availability

The original contributions presented in the study are included in the article/Supplementary material, further inquiries can be directed to the corresponding authors.
