# Supplementary material for: Cerebrospinal fluid markers and magnetic resonance imaging lesion volume predicting relapse in canine meningoencephalitis of unknown origin
Source: Front Vet Sci. 2026 Feb 10;13:1733620. doi: 10.3389/fvets.2026.1733620 (PMC12930635; doi:10.3389/fvets.2026.1733620)
Supplement: Supplementary file 1 [file Table_1.DOCX]

**Supplemental table 1 (S1):** Comparison of magnet resonance imaging (MRI) and clinical parameters between the initial MRI and the different follow up MRI examination

|  | MRI No1 | MRI No2 | MRI No3 | MRI No4 | p-value (post hoc comparison*) |
| --- | --- | --- | --- | --- | --- |
| Total lesion volume, absolute (mm³)  median (min-max), n |  |  |  |  |  |
| T2w | 3058.40  (450.00 - 18681.00), n = 35 | 1008.00  (0.00 - 7956.00) n = 35 | 1584.00  (0.00 - 8388.00), n = 13 | 2340.00  (2196.00 - 2664.00), n = 3 | p = 0.0135  (No1 vs. No2: p = 0.0110) |
| FLAIR | 4200.00  (216.00 - 24537), n = 33 | 870  (0.00 - 7560.00), n = 32 | 792.00  (0.00 - 8532.00), n = 13 | 2322.00  (1728.00 - 2916.00), n = 2 | p < 0.0001  (No1 vs. No2: p < 0.0001; No1 vs. No3: p = 0.0113) |
| T1w contrast enhancement | 900  (0.00 - 22330.70), n = 35 | 0.00  (0.00 - 2872.00), n = 32 | 0.00  (0.00 - 2052.00), n = 11 | 4514.40  (0.00 - 9028.80), n = 2 | p < 0.0001  (No1 vs. No2: p < 0.0001; No1 vs. No3: p = 0.0162) |
| Total lesion volume, relative (%)  median (min-max), n |  |  |  |  |  |
| T2w | 3.27 (0.50 - 18.52), n = 35 | 1.30 (0.00 - 9.54), n = 35 | 1.98 (0.00 - 12.55), n = 13 | 2.80 (2.26 - 2.83), n = 3 | p = 0.0090,  (No1 vs. No2: p. = 0.0069) |
| FLAIR | 4.37 (0.33 - 24.62), n = 33 | 1.14 (0.00 - 11.79), n = 32 | 1.32 (0.00 - 12.77), n = 13 | 2.58 (2.07 - 3.10), n = 2 | p < 0.0001,  (No1 vs. No2: p < 0.0001; No1 vs. No3: p = 0.0231) |
| T1w contrast enhancement | 1.09 (0.00- 22.13), n= 35 | 0.00 (0.00- 3.51), n= 32 | 0.00 (0.00- 1.44), n= 11 | 4.41 (0.00- 10.83), n= 2 | p < 0.0001,  (No1 vs. No2: p < 0.0001; No1 vs. No3: p= 0.0239) |
| Number of lesions  median (min-max), n |  |  |  |  |  |
| T2w | 2.00 (1.00 - 7.00), n = 35 | 2.00 (0.00 - 7.00), n = 35 | 2.00 (0.00 - 6.00), n = 13 | 4.00 (3.00 - 5.00), n = 3 | p > 0.05 |
| FLAIR | 3.00 (1.00 - 7.00), n = 33 | 1.00 (0.00 - 6.00), n = 32 | 1.00 (0.00 - 6.00), n = 13 | 3.50 (3.00 - 4.00), n = 2 | p = 0.0063  (No1 vs. No2: p = 0.0092) |
| T1w contrast enhancement | 3.00 (0.00 - 9.00), n = 35 | 0.00 (0.00 - 3.00), n = 32 | 0.00 (0.00 - 4.00), n = 11 | 1.00 (0.00 - 2.00), n = 2 | p < 0.0001  (No1 vs. No2: p < 0.0001) |
| New lesion compared to last MRI (yes) |  |  |  |  |  |
| T2w |  | n= 10/35 (28,57 %) | n= 6/13 (46,15 %) | n= 2/3 (66,67 %) | p < 0.0001 |
| FLAIR |  | n= 6/32 (18.75 %) | n= 6/13 (46,15 %) | n= 1/2 (50,00 %) | p < 0.0001 |
| T1w contrast enhancement |  | n= 0/32 (0.00 %) | n= 3/11 (27,27 %) | n= 1/2 (50,00 %) | p < 0.0001 (No2 vs. No3: p = 0.0134) |
| Interthalamic adhesion size (mm) | 8.50 (5.84 - 9.82), n = 30 | 7.54 (5.48 - 9.56), n = 35 | 7.44 (5.15 - 9.56), n = 13 | 8.70 (5.30 - 9.01), n = 3 | p > 0.05 |
| Brain volume (mm³)  median (min-max), n | 87648.70 (54172.20 - 145898.00), n = 35 | 88360.80 (54369.70 - 142544.00), n = 35 | 76728.20 (53279.50 - 141571.00), n = 13 | 93986.60 (83296.70 - 96853.70), n = 3 | p > 0.05 |
| Lesion in cerebellum (yes) |  |  |  |  |  |
| T2w | n = 10/35 (28,57%) | n = 3/35 (8.57 %) | n = 1/13 (7.69 %) | n = 0/3 (0.00 %) | p > 0.05 |
| FLAIR | n = 10/33 (30.30 %) | n = 4/32 (12.50 %) | n = 2/13 (15.38 %) | n = 1/2 (50.00 %) | p > 0.05 |
| T1w contrast enhancement | n = 12/35 (34.29 %) | n = 1/32 (3.13 %) | n = 1/11 (9.09 %) | n = 0/2 (0.00 %) | p = 0.0021 |
| Lesion in brainstem (yes) |  |  |  |  |  |
| T2w | n = 19/35 (54.29 %) | n = 8/35 (22.86 %) | n = 4/13 (30.77 %) | n = 2/3 (66.67 %) | p > 0.05 |
| FLAIR | n = 18/33 (54.55 %) | n = 7/32 (21.88 %) | n = 3/13 (23.08 %) | n = 0/2 (0.00 %) | p > 0.05 |
| T1w contrast enhancement | n = 19/35 (54.29 %) | n = 5/32 (15.63 %) | n = 1/11 (9.09 %) | n = 0/2 (0.00 %) | p > 0.05 |
| Lesion in forebrain (yes) |  |  |  |  |  |
| T2w | n = 28/35 (80.00 %) | n = 27/35 (77.14%) | n = 10/13 (76.92 %) | n = 3/3 (100.00 %) | p > 0.05 |
| FLAIR | n = 27/33 (81.82 %) | n = 22/32 (68.75 %) | n = 7/13 (53.85 %) | n = 2/2 (100.00 %) | p > 0.05 |
| T1w contrast enhancement | n = 20/35 (57.14 %) | n = 3/32 (9.38 %) | n = 1/11 (9.09 %) | n = 1/2 (50.00 %) | p < 0.0001 |
| Lesion in cingulate gyrus (yes) |  |  |  |  |  |
| T2w | n = 6/35 (17.14 %) | n = 1/35 (2.86 %) | n = 1/13 (7.69 %) | n = 0/3 (0.00 %) | p > 0.05 |
| FLAIR | n = 11/33 (33.33 %) | n = 3/32 (9.38 %) | n = 1/13 (7.69 %) | n = 0/2 (0.00 %) | p > 0.05 |
| T1w contrast enhancement | n = 5/35 (14.29 %) | n = 0/32 (0.00 %) | n = 0/11 (0.00 %) | n = 0/2 (0.00 %) | p > 0.05 |
| Lesion in hippocampus (yes) |  |  |  |  |  |
| T2w | n = 7/35 (20.00 %) | n = 2/35 (5.71 %) | n = 1/13 (7.69 %) | n = 0/3 (0.00 %) | p > 0.05 |
| FLAIR | n = 9/33 (27.27 %) | n = 1/32 (3.13 %) | n = 1/13 (7.69 %) | n = 0/2 (0.00 %) | p > 0.05 |
| T1w contrast enhancement | n = 4/35 (11.43 %) | n = 0/32 (0.00 %) | n = 0/11 (0.00 %) | n = 0/3 (0.00 %) | p > 0.05 |
| Lesion in piriform lobe (yes) |  |  |  |  |  |
| T2w | n = 12/35 (34.29 %) | n = 10/35 (28.57 %) | n = 5/13 (38.46 %) | n = 1/3 (33.33 %) | p > 0.05 |
| FLAIR | n = 12/33 (36.36 %) | n = 6/32 (18.75 %) | n = 4/13 (30.77 %) | n = 7/2 (0.00 %) | p > 0.05 |
| T1w contrast enhancement | n = 3/35 (8.57 %) | n = 0/32 (0.00 %) | n = 0/11 (0.00 %) | n = 1/2 (50.00 %) | p > 0.05 |
| Lesion in frontal lobe (yes) |  |  |  |  |  |
| T2w | n = 15/35 (42.86 %) | n = 9/35 (25.71 %) | n = 4/13 (30.77 %) | n = 2/3 (66.67 %) | p > 0.05 |
| FLAIR | n = 19/33 (57.58 %) | n = 6/32 (18.75 %) | n = 4/13 (30.77 %) | n = 2/2 (100.00 %) | p = 0.0166 |
| T1w contrast enhancement | n = 18/35 (51.43 %) | n = 4/32 (12.50 %) | n = 2/11 (18.18 %) | n = 1/2 (50.00 %) | p > 0.05 |
| Lesion meninges (yes) |  |  |  |  |  |
| T2w | n = 20/35 (57.14 %) | n = 10/35 (28.57 %) | n = 6/13 (46.15 %) | n = 1/3 (33.33 %) | p = 0.0326 |
| FLAIR | n = 12/33 (36.36 %) | n = 3/32 (9.38 %) | n = 3/13 (23.08 %) | n = 0/2 (0.00 %) | p = 0.0102 |
| T1w contrast enhancement | n = 9/35 (25.71 %) | n = 1/32 (3.13 %) | n = 1/11 (9.09 %) | n = 0/2 (0.00 %) | p = 0.0406 |
| Loss of sulci (yes) |  |  |  |  |  |
| T2w | n = 27/35 (77.14 %) | n = 3/35 (8.57 %) | n = 2/13 (15.38 %) | n = 0/3 (0.00 %) | p < 0.0001 |
| FLAIR | n = 26/33 (78.79 %) | n = 4/32 (12.50 %) | n = 2/13 (15.38 %) | n = 1/2 (50.00 %) | p < 0.0001 |
| T1w contrast enhancement | n = 14/35 (40.00 %) | n = 1/32 (3.13 %) | n = 1/11 (9.09 %) | n = 0/2 (0.00 %) | p = 0.0006 |
| Duration after diagnosis (days)  median (min-max), n | 0 | 112.50 (65.00 - 443.00),  n = 35 | 360.50 (223.00 – 1363.00),  n = 13 | 483.00 (301.00 - 1528.00),  n = 3 | p < 0.0001 (No2 vs. No3: < 0.0001; No2 vs. No4: p = 0.0175) |
| NDS  median (min-max), n | 4.00 (1 - 10), n = 35 | 1.00 (0.00 - 5.00), n = 33 | 1.00 (0.00 - 4.00), n = 11 | 3.00 (3.00), n = 1 | p < 0.0001  (No1 vs. No2: p < 0.0001; No1 vs. No3: p = 0.0005) |
| CSF WBC (cells/3µl)  median (min-max), n | 39.00 (0.00 - 4064.00),  n = 34 | 4.00 (0.00 - 46.00), n = 33 | 7.00 (0.00 - 35.00), n = 9 | 6.00 (2.00 - 14.00), n = 3 | p < 0.0001, (No1 vs. No2: p < 0.0001; No1 vs. No3: p = 0.0393) |
| CSF lymphocytes %  median (min-max), n | 72.41 (8.00 - 100.00), n = 29 | 78.17 (16.00 - 100.00),  n = 24 | 69.12 (11.00 - 100-00), n = 6 | 83.33 (70.00 - 100.00), n = 3 | p > 0.05 |
| CSF neutrophilic granulocytes %  median (min-max), n | 4.76 (0.00 - 84.00), n = 35 | 0.00 (0.00 - 83.00), n = 35 | 0.00 (0.00 - 17.30), n = 13 | 4.00 (0.00 - 16.66), n = 3 | p = 0.0274 |
| CSF macrophages/large monocytes %  median (min-max), n | 8.00 (0.00 - 47.50), n = 35 | 0.00 (0.00 - 100.00), n = 35 | 0.00 (0.00 - 50.00), n = 13 | 0.00 (0.00 - 26.00), n = 3 | p > 0.05 |
| CSF protein (mg/dl)  median (min-max), n | 43.40 (12.86 - 243.35),  n = 24 | 16.82 (9.30 - 75.44), n = 32 | 27.76 (22.62 - 46.12), n = 8 | 30.83 (15.51 - 44.38), n = 3 | p < 0.0001, (No1 vs. No2: p < 0.0001; No2 vs. No3: p = 0.0036) |
| CSF albumin (mg/dl)  median (min-max), n | 26.00 (3.01 - 85.85), n = 30 | 10.14 (4.00 - 26.49), n = 30 | 20.33 (11.05 - 28.86), n = 7 | 7.86 (4.50 - 28.40), n = 3 | p = 0.0012, (No1 vs. No2: p = 0.0031; No2 vs. No3: p = 0.0133) |
| CSF QAlb  median (min-max), n | 7.32 (1.00 - 21.14), n = 16 | 2.85 (1.20 - 5.83), n = 27 | 4.76 (2.58 - 7.02), n = 5 | 4.38 (2.00 - 6.76), n = 2 | p = 0.0014, (No1 vs. No2: p = 0.0019) |
| Prednisone (mg/kg/day), n | 1.04 (0.84 - 3.28), n = 35 | 0.71 (0.00 - 1.25), n = 35 | 0.49 (0.00 - 2.66), n = 12 | 0.78 (0.00 - 1.11), n = 3 | p <0.0001, (No1 vs. No2: p < 0.0001; No1 vs. No3: p = 0.0019) |
| add on medication (yes) | n = 16/35 (45.71 %) | n = 26/35 (74.29 %) | n = 9/13 (69.23 %) | n = 2/3 (66.67 %) | p > 0.05 |

Comparison of magnet resonance imaging (MRI) and clinical parameters between different follow up MRI examination, grouped by the sequence of MRI follow-up examinations (MRI No1 - 4), n = 35 dogs were included.

CSF: cerebrospinal fluid; T2w: T2 weighted; FLAIR: fluid attenuation inversion recovery; T1w: T1 weighted; NDS: Neurodisability score; WBC: white blood cell count; QAlb: albumin CSF to serum ratio; n: number

*If no results of post hoc tests are given, the results of post hoc testing are p > 0.05
