# Supplementary material for: Cerebrospinal fluid markers and magnetic resonance imaging lesion volume predicting relapse in canine meningoencephalitis of unknown origin
Source: Front Vet Sci. 2026 Feb 10;13:1733620. doi: 10.3389/fvets.2026.1733620 (PMC12930635; doi:10.3389/fvets.2026.1733620)
Supplement: Supplementary file 2 [file Table_2.DOCX]

**Supplemental table 2: (S2):** Comparison of magnet resonance imaging (MRI) and clinical parameters between different follow up MRI examination, grouped by the clinical indication for MRI

|  | MRI No1 (=initial MRI for diagnosis of MUO) | MRI, routine control | MRI, at time of relapse | MRI, after relapse | p-value (post hoc comparison*) |
| --- | --- | --- | --- | --- | --- |
| Total lesion volume, absolute (mm³)  median (min-max), n |  |  |  |  |  |
| T2w | 3058.40  (450.00 - 18681.00), n = 35 | 1116.00  (0.00 - 7956.00), n = 41 | 2502.05  (1404.00 - 8388.00), n = 4 | 2118.00 (612.00 - 5412.00), n = 6 | p = 0.0052 (No1 vs. routine: p = 0.0048) |
| FLAIR | 4200.00 (216.00 - 24537.60), n = 33 | 684.00 (0.00 - 7560.00),  n = 38 | 3318.00 (1440 - 8532.00),  n = 4 | 2700.00 (1728.00 - 4950.00), n = 5 | p < 0.0001, (No1 vs. routine: p < 0.0001; routine vs. after relapse: p = 0.0360) |
| T1w contrast enhancement | 900.00 (0.00 - 22330.70),  n = 35 | 0.00 (0.00 - 2872.00), n = 37 | 81.00 (0.00 - 410.40), n = 4 | 464.75 (0.00 - 9028.80),  n = 4 | p < 0.0001, (No1 vs. routine: p < 0.0001) |
| Total lesion volume, relative (%)  median (min-max), n |  |  |  |  |  |
| T2w | 3.27 (0.50 - 18.52), n = 35 | 1.49 (0.00 - 9.54), n = 41 | 2.58 (1.30 - 12.55), n = 4 | 3.18 (0.91 - 8.40), n = 6 | p = 0.0023 (No1 vs. routine: p = 0.0025) |
| FLAIR | 4.37 (0.33 - 24.62), n = 33 | 0.61 (0.00 - 11.79), n = 38 | 3.41 (1.33 - 12.77), n = 4 | 3.82 (2.07 - 7.68), n = 5 | p < 0.0001, (No1 vs. routine: p < 0.0001; routine vs. after relapse: p = 0.0232) |
| T1w contrast enhancement | 1.09 (0.00 - 22.13), n = 35 | 0.00 (0.00 - 3.51), n = 37 | 0.08 (0.00 - 0.61), n = 4 | 0.80 (0.00 - 10.83), n = 4 | p < 0.0001, (No1 vs. routine: p < 0.0001; routine vs. after relapse: p = 0.0038) |
| Number of lesions  median (min-max), n |  |  |  |  |  |
| T2w | 2.00 (1.00 - 7.00), n = 35 | 2.00 (0.00 - 7.00), n = 40 | 4.00 (2.00 - 5.00), n = 4 | 3.00 (1.00 - 5.00), n = 6 | p > 0.05 |
| FLAIR | 3.00 (1.00 - 7.00), n = 33 | 1.00 (0.00 - 6.00), n =38 | 4.00 (2.00 - 6.00), n = 4 | 3.00 (2.00 - 4.00), n = 5 | p < 0.0001, (No1 vs. routine: p = 0.0004; routine vs. relapse: p = 0.0399) |
| T1w contrast enhancement | 3.00 (0.00 - 9.00), n = 35 | 0.00 (0.00 - 3.00), n = 36 | 0.50 (0.00 - 4.00), n = 4 | 2.50 (1.00 - 4.00), n = 4 | p < 0.0001, (No1 vs. routine: p < 0.0001; routine vs. after relapse: p = 0.0360) |
| New lesion (yes) |  |  |  |  |  |
| T2w |  | n = 11/41 (26.83 %) | n = 2/4 (50.00 %) | n = 5/6 (83.33 %) | p < 0.0001 (after relapse vs. Routine: p = 0.0029) |
| FLAIR |  | n = 6/38 (15.79 %) | n = 2/4 (50.00 %) | n = 5/5 (100.00 %) | p < 0.0001 (after relapse vs. Routine: p = 0.0001) |
| T1w contrast enhancement |  | n = 0/37 (0.00 %) | n = 1/4 (25.00 %) | n = 3/4 (75.00 %) | p < 0.0001(after relapse vs. Routine: p < 0.0001) |
| Interthalamic adhesion size (mm) | 8.50 (5.84 - 9.82), n = 30 | 7.54 (5.15 - 9.56), n = 41 | 8.33 (5.84 - 8.78), n = 4 | 6.56 (5.30 - 8.23), n = 6 | p = 0.0255 (No1 vs. after relapse: p = 0.0397) |
| Brain volume (mm³)  median (min-max), n | 87648.70 (54172.20 - 145898.00), n = 35 | 88744.20 (54369.70 - 142544.00), n = 41 | 97007.30 (66783.70 - 107578.00), n = 4 | 67052.00 (53279.50 - 83296.70), n = 6 | p > 0.05 |
| Duration after diagnosis (days)  median (min-max), n | 0 | 120.00 (65.00 - 443.00),  n = 41 | 312.50 (183.00 - 483.00),  n = 4 | 1123.50 (329.00 - 1528.00), n= 6 | p = 0.0017 (Routine vs. AR: p = 0.0060) |
| NDS  median (min-max), n | 4.00 (1.00 - 10.00), n = 35 | 1.00 (0.00 - 3.00), n = 37 | 3.00 (1.00 - 5.00), n = 3 | 3.00 (2.00 - 4.00), n = 5 | p < 0.0001, (No1 vs. routine: p < 0.0001; routine vs. after relapse: p = 0.0019) |
| CSF WBC (cells)  median (min-max), n | 39.00 (0.00 - 4064.00),  n = 34 | 3.50 (0.00 - 46.00), n = 36 | 4.50 (0.00 - 30.00), n = 4 | 8.00 (5.00 - 17.00), n = 5 | p < 0.0001, (No1 vs. routine: p < 0.0001) |
| CSF lymphocytes %  median (min-max), n | 72.41 (8.00 - 100.00), n = 29 | 73.50 (16.00 - 100.00),  n = 26 | 87.50 (83.33 - 100.00), n = 3 | 74.28 (11.00 - 82.69), n = 4 | p > 0.05 |
| CSF neutrophilic granulocytes %  median (min-max), n | 4.76 (0.00 - 84.00), n = 35 | 0.00 (0.00 - 83.00), n = 41 | 0.00 (0.00 - 16.66), n = 4 | 0.00 (0.00 -17.30), n = 6 | p = 0.0468 |
| CSF macrophages/large monocytes %  median (min-max), n | 8.00 (0.00 - 47.50), n = 35 | 0.00 (0.00 - 100.00), n = 41 | 0.00 (0.00), n = 4 | 2.00 (0.00 - 26.00), n = 6 | p = 0.0482 |
| CSF protein (mg/dl)  median (min-max), n | 43.40 (12.86 - 243.35),  n = 34 | 18.51 (9.30 - 46.12), n = 35 | 18.93 (10.78 - 44.38), n = 3 | 30.83 (13.31 - 75.44), n = 5 | p = 0.0002 (No1 vs. routine: p = 0.0002) |
| CSF albumin (mg/dl)  median (min-max), n | 26.00 (3.01 - 85.85), n = 30 | 11.05 (4.33 - 28.86), n = 33 | 11.74 (6.85 - 28.40), n = 3 | 11.55 (4.00 - 21.73), n = 4 | p = 0.0136 (No1 vs. routine: p = 0.0094) |
| CSF QAlb  median (min-max), n | 7.32 (1.00 - 21.14), n = 16 | 2.86 (1.20 - 7.02), n = 28 | 3.08 (1.54 - 6.76), n = 3 | 4.49 (2.56 - 6.69), n = 3 | p = 0.0055 (No1 vs. routine: p = 0.0036) |
| Prednisone (mg/kg/day), n | 1.04 (0.84 - 3.28), n = 35 | 0.62 (0.00 - 1.25), n = 41 | 0.93 (0.28 - 1.11), n = 4 | 0.51 (0.00 - 2.66), n = 5 | p < 0.0001 (No1 vs. Routine: p < 0.0001) |
| add on medication (yes) | n = 16/35 (45.71 %) | n = 30/41 (73.17 %) | n = 3/4 (75.00 %) | n = 4/6 (66.67 %) | p > 0.0853 |

Comparison of magnet resonance imaging (MRI) and clinical parameters between different follow up MRI examination, grouped by the clinical indication for MRI (initial diagnosis of MUO, routine control MRI without current signs of relapse, due to relapse of clinical signs, control after relapse of clinical signs), n = 35 dogs were included.

CSF: cerebrospinal fluid; T2w: T2 weighted; FLAIR: fluid attenuation inversion recovery; T1w: T1 weighted; NDS: Neurodisability score; WBC: white blood cell count; QAlb: albumin CSF to serum ratio; n: number; No1: initial MRI for diagnosis of MUO; routine: routine control MRI.

*If no results of post hoc tests are given, the results of post hoc testing are p > 0.05
