# Supplementary material for: Cerebrospinal fluid markers and magnetic resonance imaging lesion volume predicting relapse in canine meningoencephalitis of unknown origin
Source: Front Vet Sci. 2026 Feb 10;13:1733620. doi: 10.3389/fvets.2026.1733620 (PMC12930635; doi:10.3389/fvets.2026.1733620)
Supplement: Supplementary file 3 [file Table_3.DOCX]

**Supplemental table 3 (S3):** Comparison of magnet resonance imaging (MRI) and clinical parameters at initial diagnosis (MRI No1) between dogs with relapse and dogs without relapse

|  | relapse | no relapse | p-value |
| --- | --- | --- | --- |
| gender | 10/17 male; 7/17 female | 8/18 male; 10/18 female | p > 0.05 |
| age at diagnosis (days)  median (min-max), n | 1787.00 (499.00 - 3775.00), n = 17 | 976.50 (134.00 - 4090.00), n = 18 | p > 0.05 |
| Weight (kg), n | 6.40 (1.50 - 31.40), n = 17 | 12.20 (1.40 - 39.80), n = 18 | p > 0.05 |
| Findings at initial diagnosis: |  |  |  |
| Total lesion volume, absolute (mm³)  median (min-max), n |  |  |  |
| T2w | 2124.00 (450.00 - 12270.00), n = 17 | 3762.00 (528.00 - 18681.00), n =18 | p > 0.05 |
| FLAIR | 3504.00 (470.40 - 11160.00), n = 17 | 5100.00 (216.00 - 24537.60), n = 16 | p > 0.05 |
| T1w contrast enhancement | 900.00 (0.00 - 10784.40), n = 17 | 1185.60 (0.00 - 22330.70), n = 18 | p > 0.05 |
| Total lesion volume, relative (%)  median (min-max), n |  |  |  |
| T2w | 3.02 (0.50 - 17.62), n = 17 | 4.14 (0.87 - 18.52), n = 18 | p > 0.05 |
| FLAIR | 4.27 (0.49 - 16.84), n = 17 | 5.96 (0.33 - 24.62), n = 16 | p > 0.05 |
| T1w contrast enhancement | 1.00 (0.00 - 15.56), n = 17 | 1.23 (0.00 - 22.13), n = 18 | p > 0.05 |
| Number of lesions  median (min-max), n |  |  |  |
| T2w | 2.00 (1.00 - 7.00), n = 17 | 2.50 (1.00 - 4.00), n =18 | p > 0.05 |
| FLAIR | 3.00 (1.00 - 6.00), n = 17 | 2.50 (1.00 - 7.00), n = 16 | p > 0.05 |
| T1w contrast enhancement | 3.00 (0.00 - 9.00), n = 17 | 2.00 (0.00 - 3.00), n = 18 | p > 0.05 |
| total brain volume (mm³), n | 82765.60 (54172.20 - 127668.00), n = 17 | 89822.35 (57170.00 - 145898.00), n = 18 | p > 0.05 |
| NDS  median (min-max), n | 4.00 (1.00 - 9.00), n =17 | 4.00 (1.00 - 10.00), n = 18 | p > 0.05 |
| CSF WBC (cells/3µl)  median (min-max), n | 35.00 (0.00 - 1776.00), n =16 | 68.50 (0.00 - 4064.00), n = 18 | p > 0.05 |
| CSF lymphocytes %  median (min-max), n | 71.00 (13.00 - 97.00), n = 13 | 74.84 (8.00 - 100.00), n = 16 | p > 0.05 |
| CSF neutrophilic granulocytes %  median (min-max), n | 3.00 (0.00 - 70.00), n = 17 | 4.88 (0.00 - 84.00), n = 18 | p > 0.05 |
| CSF macrophages/large monocytes %  median (min-max), n | 8.00 (0.00 - 47.50), n = 17 | 8.00 (0.00 - 42.00), n = 18 | p > 0.05 |
| CSF protein (mg/dl)  median (min-max), n | 45.67 (12.86 - 210.98), n = 16 | 41.31 (14.60 - 243.35), n = 18 | p > 0.05 |
| CSF albumin (mg/dl)  median (min-max), n | 29.98 (3.01 - 85.85), n = 14 | 22.05 (5.08 - 84.37), n = 16 | p > 0.05 |
| CSF QAlb  median (min-max), n | 6.20 (1.00 - 19.86), n = 7 | 7.48 (3.15 - 21.14), n = 9 | p > 0.05 |
| Prednisone (mg/kg/day), n | 1.00 (0.84 - 1.70), n = 17 | 1.08 (0.84 - 3.28), n = 18 | p > 0.05 |
| add on medication (yes) | n = 8/17 (47.06 %) | n = 8/18 (44.44 %) | p > 0.05 |

Comparison of magnet resonance imaging (MRI) and clinical parameters at initial diagnosis (MRI No1) between dogs with relapse and dogs without relapse, n = 35 dogs were included.

CSF: cerebrospinal fluid; T2w: T2 weighted; FLAIR: fluid attenuation inversion recovery; T1w: T1 weighted; NDS: Neurodisability score; WBC: white blood cell count; QAlb: albumin CSF to serum ratio; n: number
