# Supplementary material for: Cerebrospinal fluid markers and magnetic resonance imaging lesion volume predicting relapse in canine meningoencephalitis of unknown origin
Source: Front Vet Sci. 2026 Feb 10;13:1733620. doi: 10.3389/fvets.2026.1733620 (PMC12930635; doi:10.3389/fvets.2026.1733620)
Supplement: Supplementary file 4 [file Table_4.DOCX]

**Supplemental table 4 (S4):** Comparison of magnet resonance imaging (MRI) and clinical parameters of only routine follow up MRI examination without relapse of clinical signs

|  | before relapse | no relapse | p-value |
| --- | --- | --- | --- |
| Weight (kg), n | 7.50 (1.90- 31.10), n = 17 | 13.05 (1.50- 38.20), n = 24 | p > 0.05 |
| total brain volume (mm³),  median (min-max), n | 83132.70 (54369.70 - 127727.00), n = 17 | 94791.55 (57361.90 – 142544.00), n = 24 | p > 0.05 |
| Total lesion volume, absolute (mm³)  median (min-max), n |  |  |  |
| T2w | 684.00 (0.00 - 7956.00), n = 17 | 2160.00 (0.00 - 6120.00), n = 24 | p = 0.0165 |
| FLAIR | 657.00 (0.00 - 3168.00), n = 16 | 684.00 (0.00 - 7560.00), n = 22 | p > 0.05 |
| T1w contrast enhancement | 0.00 (0.00 - 2029.20), n = 16 | 0.00 (0.00 - 2872.80), n = 21 | p > 0.05 |
| Total lesion volume, relative (%)  median (min-max), n |  |  |  |
| T2w | 0.76 (0.00 - 6.22), n = 17 | 1.82 (0.00 - 9. 54), n = 24 | p = 0.0213 |
| FLAIR | 0.71 (0.00 - 5.49), n = 16 | 0.61 (0.00 - 11.79), n = 22 | p > 0.05 |
| T1w contrast enhancement | 0.00 (0.00 - 3.51), n = 16 | 0.00 (0.00 - 2.01), n = 21 | p > 0.05 |
| Number of lesions  median (min-max), n |  |  |  |
| T2w | 2.00 (0.00 - 6.00), n = 17 | 2.00 (0.00 - 7.00), n = 24 | p > 0.05 |
| FLAIR | 2.00 (0.00 - 6.00), n = 16 | 1.00 (0.00 - 6.00), n = 22 | p > 0.05 |
| T1w contrast enhancement | 0.00 (0.00 - 3.00), n = 16 | 0.00 (0.00 - 2.00), n = 21 | p > 0.05 |
| New lesion (yes) |  |  |  |
| T2w | n = 5/17 (29.41 %) | n = 6/24 (25.00 %) | p > 0.05 |
| FLAIR | n = 1/16 (6.25 %) | n = 5/22 (22.73 %) | p > 0.05 |
| T1w contrast enhancement | n = 0/16 (0.00 %) | n = 0/21 (0.00 %) | p > 0.05 |
| NDS  median (min-max), n | 0.50 (0.00 - 3.00), n = 16 | 1.00 (0.00 - 3.00), n = 21 | p > 0.05 |
| CSF WBC (cells/3µl)  median (min-max), n | 3.00 (0.00 - 14.00), n = 16 | 4.00 (0.00 - 46.00), n = 20 | p > 0.05 |
| CSF lymphocytes %  median (min-max), n | 95.00 (55.55 - 100.00), n = 12 | 55.88 (16.00 - 100.00), n = 14 | p = 0.0329 |
| CSF neutrophilic granulocytes %  median (min-max), n | 0.00 (0.00 - 33.33), n = 17 | 0.00 (0.00 - 83.00), n = 24 | p > 0.05 |
| CSF macrophages/large monocytes %  median (min-max), n | 0.00 (0.00 - 33.33), n = 17 | 0.00 (0.00 - 100.00), n = 24 | p > 0.05 |
| CSF protein (mg/dl)  median (min-max), n | 23.06 (11.53 - 46.12), n = 17 | 16.59 (9.30 - 37.47), n = 18 | p > 0.05 |
| CSF albumin (mg/dl)  median (min-max), n | 11.72 (5.15 - 28.86), n = 15 | 8.19 (4.33 - 26.49), n = 18 | p = 0.0222 |
| CSF QAlb  median (min-max), n | 3.72 (1.38 - 7.02), n = 12 | 2.31 (1.20 - 5.83), n = 16 | p > 0.05 |
| Prednisone at the time of MRI (mg/kg/day), n | 0.72 (0.00 - 1.25), n = 17 | 0.52 (0.00 - 1.09), n = 24 | p > 0.05 |
| add on medication at the time of MRI (yes) | n = 13/17 (76.47 %) | n = 17/24 (70.83 %) | p > 0.05 |
| medication decreased (yes) | n = 8/17 (47.06 %) | n = 19/24 (79.17 %) | p = 0.0476 |

Comparison of Magnet resonance imaging (MRI) and clinical parameters of only routine follow up MRI examination without relapse of clinical signs, n = 41 MRIs of n = 31 dogs were included

CSF: cerebrospinal fluid; T2w: T2 weighted; FLAIR: fluid attenuation inversion recovery; T1w: T1 weighted; NDS: Neurodisability score; WBC: white blood cell count; QAlb: albumin CSF to serum ratio; n: number
